# The Morphological Diversity of Dragon Lacewing Larvae (Nevrorthidae, Neuroptera) Changed More over Geological Time Scales Than Anticipated

**DOI:** 10.3390/insects14090749

**Published:** 2023-09-06

**Authors:** Laura Mengel, Simon Linhart, Gideon T. Haug, Thomas Weiterschan, Patrick Müller, Christel Hoffeins, Hans-Werner Hoffeins, Viktor Baranov, Carolin Haug, Joachim T. Haug

**Affiliations:** 1 Faculty of Biology, Biocenter, Ludwig-Maximilians-Universität München (LMU Munich), Großhaderner Str. 2, 82152 Planegg-Martinsried, Germany; la.mengel@campus.lmu.de (L.M.); s.linhart@campus.lmu.de (S.L.); gideon.haug@palaeo-evo-devo.info (G.T.H.); joachim.haug@palaeo-evo-devo.info (J.T.H.); 2 Independent Researcher, 64739 Höchst im Odenwald, Germany; thomas.weiterschan@web.de; 3 Independent Researcher, 66482 Zweibrücken, Germany; pat14789@web.de; 4 Independent Researcher, 22149 Hamburg, Germany; chw.hoffeins@googlemail.com (C.H.); hoffeins@aol.com (H.-W.H.); 5 Estación Biológica de Doñana-CSIC, 41092 Sevilla, Spain; viktor.baranov@ebd.csic.es; 6 GeoBio-Center at LMU, Richard-Wagner-Str. 10, 80333 München, Germany

**Keywords:** Nevrorthidae, Neuroptera, Burmese amber, quantitative morphology, morphometrics

## Abstract

**Simple Summary:**

Nevrorthidae is the group of dragon lacewings, an ingroup of lacewings (Neuroptera). Nevrorthidae has often been considered a relic group. Today, dragon lacewings are known in a few regions with rather large distances between them, with some species occurring in southern Europe, Japan, Australia, and one in China. Fossils in amber from the Baltic region (c. 35–40 million years old) and Myanmar (c. 100 million years old) support the idea that this distribution is only a remnant of an originally larger distribution. Larvae of the group are slender and elongated and live mostly in water. Yet, larvae are in fact very rare. So far, only slightly more than 30 larval specimens have been depicted in the literature, including all extant and fossil larvae. Here, we report numerous additional specimens, including extant larvae, but also larvae in Baltic and Myanmar amber. Together with the already known ones, this sums up to over 100 dragon lacewing larvae. We used quantitative methods to study the morphology of these larvae and compared these results over time to identify changes in the diversity. Although there are now more specimens in the data set, it is still unbalanced; for example, newly hatched larvae (several dozen specimens) are only known from Baltic amber. We expected little change in the morphology of the larvae over geological time, as indicated by earlier studies. However, on the contrary, there are morphologies present in fossil larvae that are now extinct. This result is similar to that for other groups of lacewings which have a relic distribution today, as also in larvae of these groups, there is a lower diversity today than in the past.

**Abstract:**

Nevrorthidae, the group of dragon lacewings, has often been considered a relic group. Today, dragon lacewings show a scattered distribution, with some species occurring in southern Europe, Japan, Australia, and one in China. The idea that this distribution is only a remnant of an originally larger distribution is further supported by fossils of the group preserved in ambers from the Baltic region (Eocene, ca. 35–40 MaBP) and Myanmar (Kachin amber, Cretaceous, ca. 100 MaBP). Larvae of the group are slender and elongated and live mostly in water. Yet, larvae are in fact very rare. So far, only slightly more than 30 larval specimens, counting all extant and fossil larvae, have been depicted in the literature. Here, we report numerous additional specimens, including extant larvae, but also fossil ones from Baltic and Kachin amber. Together with the already known ones, this sums up to over 100 specimens. We analysed quantitative aspects of the morphology of these larvae and compared them over time to identify changes in the diversity. Despite the enriched sample size, the data set is still unbalanced, with, for example, newly hatched larvae (several dozen specimens) only known from the Eocene. We expected little change in larval morphology over geological time, as indicated by earlier studies. However, on the contrary, we recognised morphologies present in fossils that are now extinct. This result is similar to those for other groups of lacewings which have a relic distribution today, as these have also suffered a loss in diversity in larval forms.

## 1. Introduction

The group Insecta experiences a general decline in diversity and abundance [1,2,3,4,5,6]. Representatives of the group such as bees, beetles, and butterflies play important roles in various processes of the ecosystems: they are pollinators, decomposers of dead organic material, and are a source of food for other larger animals [1]. Therefore, a loss of the diversity within Insecta has a negative effect on ecosystem functioning. It is of major importance to investigate evolutionary processes leading to diversifications and losses in order to improve our understanding of such processes and potentially how to alter them.

The diversity of Insecta basically is that of its ingroup Holometabola, or better of several of the lineages within the latter. For looking at historical losses, the holometabolan group Neuroptera, the group of lacewings, is especially interesting as it is generally understood as having been part of the early diversification of Holometabola, but having declined after the Mesozoic.

As recently demonstrated, this loss is in fact challenging to show quantitatively [7]. Yet, applying quantitative morphology to the larval forms could indeed demonstrate such a loss [7], but also showed that this loss affected certain lineages much more than others.

Fossils of larvae of lacewings can luckily be well recognised as such [8,9,10,11,12,13,14,15,16,17,18,19,20,21,22,23,24,25,26], especially due to their prominent forward-projecting mouthparts [27,28]. This specific morphology allows us to recognise fossil lacewing larvae quite frequently, providing a sound basis for quantitative comparisons [29,30,31].

One neuropteran ingroup seemingly rather unaffected, at least in certain aspects, is Nevrorthidae, the group of dragon lacewings. The exact relationships within Neuroptera are still a matter of debate, but dragon lacewings seem to represent an early offshoot, either as sister group to all other lacewings or as part of the second branch within the group Osmyloidea [32,33,34,35,36,37,38,39,40,41,42]. The idea of dragon lacewings representing an early branch is supported by the fact that the oldest known fossil larva is that of a dragon lacewing [43].

The morphology of dragon lacewings, including that of the larvae, seemed more or less unchanged since the Cretaceous (a phenomenon fairly common in some groups of Holometabola across all life stages, see [44]). This result is partly surprising as the group is understood as a mere relict group. This status is based on the fact that the fossils are known from places in which they do not occur today (Germany, Myanmar, Ukraine [45,46,47]); species numbers do not provide such an impression, with more extant than fossil species (19 formally described extant species [48]; 10 formally described fossil species [49]). Yet, the record of dragon lacewing larvae is rather incomplete, and very recent findings have expanded not only the range of the fossils, but also demonstrated that fossil dragon lacewing larvae can appear quite different from their modern counterparts [43].

We here report many new fossil lacewing larvae and provide a quantitative morphological comparison of this much-expanded data set. Our findings partly contradict common expectations.

## 2. Materials and Methods

### 2.1. Materials

The data set for the comparison presented here is based on literature data and newly studied fossil and extant specimens of dragon lacewing larvae. Literature data are based on an earlier overview [50] summarising earlier sources of extant [48,51,52,53,54,55,56,57,58,59,60,61,62,63,64,65,66,67,68,69,70], (https://www.mdfrc.org.au/bugguide/, accessed on 2 July 2018) and fossil dragon lacewing larvae [46,47,61,62,71,72,73,74]. We provide additional sources from the literature (see below).

New directly studied material came from various sources. Fossil specimens are all preserved in amber, with a total of 69 specimens in 23 pieces, of which 55 specimens (in nine pieces) are preserved in Eocene Baltic amber (about 40–35 million years old) and 14 specimens (in 14 pieces) are preserved in Cretaceous Kachin amber, Myanmar (about 100 million years old [75,76,77]). Baltic amber pieces come from the collections of the Natural History Museum Denmark, Copenhagen (NHMD), the Senckenberg Naturmuseum Frankfurt (SMF), the Palaeo-Evo-Devo Research Group Collection of Arthropods, Ludwig-Maximilians-Universität München (PED), and the collection of two of the authors (CH and HWH: CCHH). Kachin amber pieces come from the collections of the Palaeo-Evo-Devo Research Group Collection of Arthropods, Ludwig-Maximilians-Universität München (PED), and the collections of two of the authors (PM: BUB; TW Weiterschan BuB). Specimens from the PED collection were purchased from Jonas Damzen, Vilnius (www.amberinclusions.eu, accessed on 5 September 2023), and on ebay.com (accessed on 5 September 2023) from three traders: burmite-miner, macro-cretaceous, and rmvveta (see also www.ambertreasure4u.com, accessed on 5 September 2023). Extant specimens, seven in total, are part of the Australian Museum Entomology Collection, Sydney.

### 2.2. Documentation Methods

All fossil specimens were documented on a Keyence VHX-6000 digital microscope (Keyence, Osaka, Japan). The built-in software automatically fused a stack of images of shifting focus into a single sharp image. Every image was recorded with several exposure times (HDR function [50]). Each resulting image is a composite image.

If possible, specimens were photographed from both sides. Illumination was cross-polarised co-axial light or unpolarised low-angle ring light with a black or white background. The images with the best contrast were then optimised with Adobe Photoshop 2023 (Version 24.0.0, Adobe, San José, CA, USA). All visible structures of the larvae were colour-marked and labelled. Corresponding structures were given the same colours.

The extant specimens were documented with a Canon EOS 650 (Canon, Tokyo, Japan) equipped with an MP-E 65 mm super-macro lens. Illumination was provided by a Yongnuo Twin Flash (Yongnuo, Shenzhen, China). Polarisers in front of the flash and the lens provided cross-polarised light. Specimens were photographed in their original storage liquid. Each image details were recorded with several frames and fused with CombineZM. Adjacent image details were merged manually in Adobe Photoshop CS2 (Adobe, San José, CA, USA). Images were further optimised (contrast, colour, removal of background) in Adobe Photoshop CS2.

### 2.3. Shape Analysis

Outlines of prominent structures were drawn digitally in Adobe Illustrator CS2 and Inkscape. These include stylets, head, and trunk (no other appendages). The stylet was artificially rotated straight (assumed line of the innermost distal point; in this case, the tip and the inner proximal point, outer proximal point as rotation point). Also, the trunk was artificially straightened. Only half of the outline was used.

Outlines (shapes) were analysed in the program package Shape [78,79]. Harmonics were set to 20, and the first harmonic was used for alignment.

In total, 10 different combinations of shapes were analysed: (1) the head capsule and stylet, (2) the stylet, (3) the head capsule, (4) the head capsule, stylet, and trunk, (5) the head capsule and trunk, (6) the head capsule, stylet, and prothorax (including the neck), (7) the head capsule and the prothorax, (8) the prothorax, (9) the trunk, and (10) the trunk without the prothorax.

## 3. Results

### 3.1. Additional Larval Representatives of Nevrorthidae Depicted in the Literature

Haug et al. [50] attempted to provide a full list of figured dragon lacewing larvae. Yet, they missed some sources, especially depictions in books. In addition, new specimens have been identified on websites and new specimens have been published since 2020. All these additional specimens are listed here chronologically. The list is an amendment to the list in Haug et al. [50]; the specimens from there (6601–6633) are not repeated here. All specimens are numbered consecutively, amending the counting from Haug et al. [50]. All numbers of Nevrorthidae have “66” in front as a code for the group Nevrorthidae when used in larger comparative studies [7].

**Figure 1:**
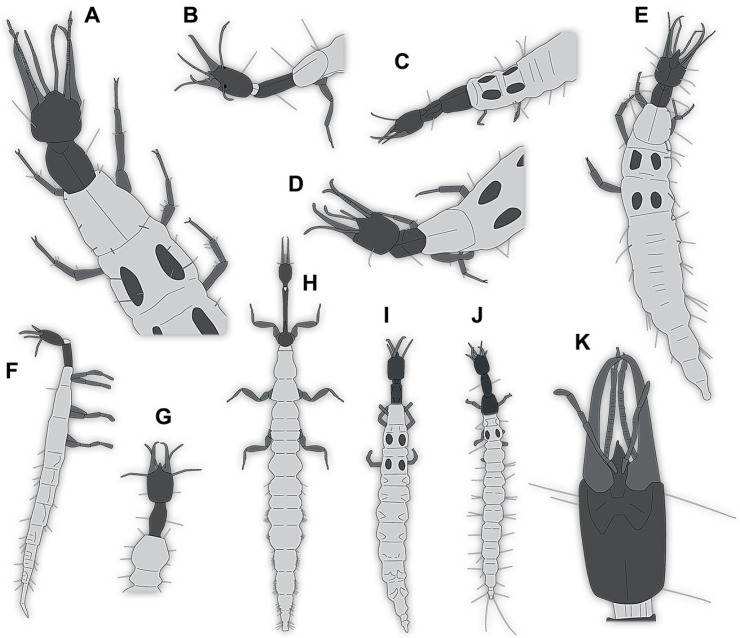
Nevrorthidae; fossil and extant immature specimens from the literature, interpretative drawings. (**A**,**C**–**E**,**H**) Fossil specimens. (**B**,**F**,**G**,**I**–**K**) Extant specimens. (**A**). Specimen 6634 preserved in Baltic amber; anterior region of the body in dorsal view; from [80]. (**B**). Specimen 6635; *Nevrorthus* (“*Neurorthus*”) *apatelius*; anterior region of the body in oblique dorsal view; from [81]. (**C**). Specimen 6636; *Rophalis relicta* preserved in Baltic amber; anterior body region in dorso-lateral view; from [81]. (**D**). Specimen 6637; anterior body region in dorsal view; from [81]. (**E**). Specimen 6638 preserved in Baltic amber; dorsal view; from [82]. (**F**,**G**). Specimen 6639; *Nevrorthus fallax*; from [83]. (**F**). Overview. (**G**). Anterior region in dorsal view. (**H**). Specimen 6640; *Girafficervix baii*; dorsal view; from [43]. (**I**). Specimen 6642; dorsal view; from https://friendsofrietvlei.co.za, accessed on 5 September 2023. (**J**). Specimen 6643; dorsal view; from https://www.waterbugblitz.org.au, accessed on 5 September 2023. (**K**). Specimen 6644; head in ventral view; from https://www.mdfrc.org.au, accessed on 5 September 2023.

(1) Janzen [80] depicted a photomicrograph (fig. 261 p. 120; Figure 1A) of a dragon lacewing larva preserved in Baltic amber (specimen 6634). The image shows only the anterior region of the body in dorsal view. According to the figure legend, the specimen is part of the collection Glink. No indication of size was provided.

(2) Scheven [81] depicted three different dragon lacewing larvae. The first specimen (6635) was an extant larva of *Nevrorthus* (“*Neurorthus*”) *apatelius* (upper left fig. p. 75; Figure 1B). Only the anterior region of the body is accessible in oblique dorsal view. No indication of size was provided.

The second and third specimens are larvae of *Rophalis relicta* preserved in Baltic amber. Of the second specimen (6636), only the anterior body region is accessible, in dorso-lateral view (lower left fig. p. 75; Figure 1C). No indication of size was provided.

Of the third specimen (6637), only the anterior body region is accessible, in dorsal view (upper right fig. p. 75; Figure 1D). No indication of size was provided.

**Figure 2:**
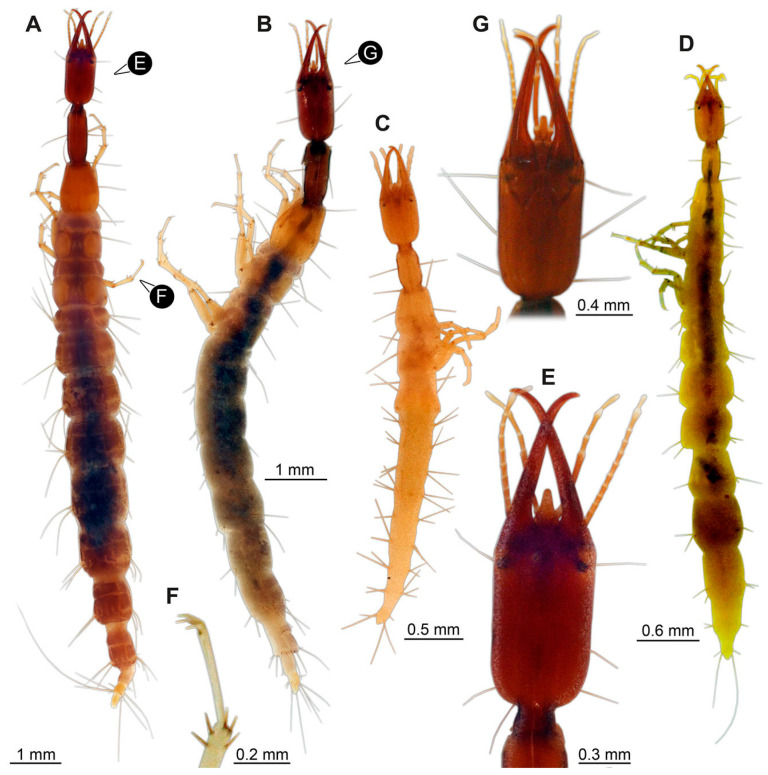
Nevrorthidae (*Austroneurorthus*); extant immature specimens. (**A**). Specimen 6645; dorsal view. (**B**). Specimen 6646; dorso-lateral view, left. (**C**). Specimen 6647; dorsal view. (**D**). Specimen 6648; dorsal view. (**E**). Close-up of head of (**A**). (**F**). Close-up of tarsus of (**A**). (**G**). Close-up of head of (**B**).

**Figure 3:**
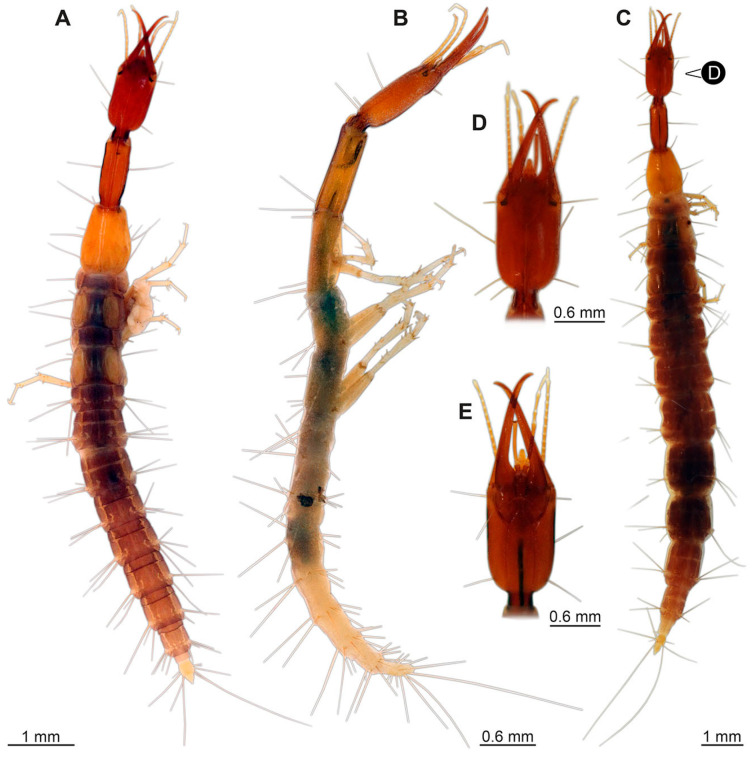
Nevrorthidae (*Austroneurorthus*); extant immature specimens. (**A**). Specimen 49; dorsal view. (**B**). Specimen 6650; lateral view, right. (**C**). Specimen 6651; dorsal view. (**D**). Close-up of dorsal side of the head of (**C**). (**E**). Close-up of ventral side of the head of specimen 6651.

(3) Kobbert [82] depicted a photomicrograph (fig. T444 p. 108; Figure 1E) of a dragon lacewing larva (specimen 6638) preserved in Baltic amber, labelled as “Neurorthidae”. The image shows the animal in dorsal view. In addition, a close-up of the anterior body was provided (fig. T444 p. 109). No indication of size was provided.
(4) Giacomino [83] depicted three photomicrographs (all on p. 118) of an extant larva (specimen 6639) of *Nevrorthus fallax*. Images include an overview in lateral view (fig. 1; Figure 1F), and the anterior head region in dorsal (fig. 2; Figure 1G) and ventral view (fig. 3). No indication of size was provided. According to the text, several specimens were available for study, but only a single one was depicted.
(5) Kobbert [84] re-figured (suppl.-fig. 203 on CD-rom) the specimen from Kobbert [82]. It was a similar view as the detail image ([82] T444 p. 109), but as a red-cyan stereo-anaglyph.
(6) Beutel et al. [85] re-figured two SEM images from Beutel et al. [67], i.e., images of specimen 6614 (both on p. 377). This includes a lateral overview (fig. 6.26.1) and a ventral view of the head region (fig. 6.26.2A).
(7) Du et al. [43] reported two fossil larvae of dragon lacewings (*Girafficervix baii*; fig. 1c, p. 3) from the Jurassic of China. The first specimen (6640) is the better preserved one (Figure 1H), almost complete in dorsal view. The second specimen (6641) is slightly less well preserved, but shows some details not available from the first specimen. Both together give a good impression of the overall morphology, which is unusual due to a long neck and an astonishing size of about 40 mm.
(8) Websites are usually not considered a “good” source for scientific research. Yet, given the scarceness of appearances of larval representatives of Nevrorthidae in the literature, we decided to also include data from three websites hosted by organisations that are likely to guarantee a long-time availability. All specimens are from the extant fauna and were retrieved on 3 June 2021.

The first specimen (6642) is seen in dorsal view (Figure 1I) on the website https://friendsofrietvlei.co.za, accessed on 5 September 2023. The website is hosted by Friends of Rietvlei, a registered non-profit organisation. The second specimen (6643) is also seen in dorsal view (Figure 1J) on the website https://www.waterbugblitz.org.au, accessed on 5 September 2023. The website is hosted by a citizen science project funded by the Australian government, named “National Waterbug Blitz”. For the third specimen (6644), the head is available in ventral view (Figure 1K) on the website https://www.mdfrc.org.au, accessed on 5 September 2023. The website is hosted by La Trobe University.

### 3.2. New Extant Larval Representatives of Nevrorthidae

(9) Specimen 6645 (accession number nsw_epa_HUNT_04Williams_r_161299) is well accessible (Figure 2A); it likely represents a final larval stage. The specimen was collected during the NSW EPA-MRHI survey, HUNT-04, in the William River at Rocky Crossing, New South Wales, Australia (32°17′7″ S, 151°48′7″ E), on 16 December 1999. Head with antennae, stylets, and labial palps. Antennae are elongated and show subdivision into numerous elements (about 12). Stylets are proximally straight, distally more curved, tapering distally. Labial palps are slightly shorter than antennae, subdivided into at least four elements apparent (Figure 2E). Trunk appendages with five major elements (coxa, trochanter, femur, tibia, tarsus; Figure 2A,F). Coxa and trochanter are short, not well accessible; femur tubular and elongated; tibia similar to femur, but slightly shorter; tarsus quite slender again and tapering distally with a pair of claws (Figure 2F). Thorax is subdivided into pro-, meso-, and metathorax, with a long cervix anteriorly. Abdomen with 11 units. The cervix and prothorax show a longitudinal groove (Figure 2A). The trunk segments posterior to the prothorax with a distinct pattern of dorsal sclerotisations. After the anterior third of the segment, a distinct fold is apparent, subdividing the dorsal region of each segment into two distinct subregions (Figure 2A). Prominent setae arise from the trunk appendage elements. Femur and tibia are distally with a set of three to five setae each. Trunk segments with prominent long setae. These arise close to the posterior end of each segment and appear to form a kind of ring around the segment.

(10) Specimen 6646 (accession number nsw_epa_HUNT_04Williams_r_161299; in the same vial as specimen 6645) is well accessible (Figure 2B); it likely represents a final larval stage. The specimen was collected during the NSW EPA-MRHI survey, HUNT-04, in the William River at Rocky Crossing, New South Wales, Australia (32°17′7″ S, 151°48′7″ E), on 16 December 1999. Overall body outline is well accessible. Head with antennae, stylets, and labial palps (Figure 2G). Antennae are elongated, subdivided into numerous elements (about 11). Stylets are proximally straight, distally curved, tapering distally. Labial palps are subdivided into five elements (Figure 2G). Trunk appendages with five major elements (coxa, trochanter, femur, tibia, tarsus; Figure 2B). Coxa is tubular and elongated; trochanter not accessible; femur similar to coxa, but thinner; tibia similar to femur, but a bit shorter; tarsus quite slender again and tapering distally with a pair of claws (Figure 2B). Thorax is subdivided into pro-, meso-, and metathorax with a long cervix anteriorly. Abdomen with 11 units. The cervix and prothorax show a longitudinal groove (Figure 2B). The trunk segments posterior to the prothorax with a distinct pattern of dorsal sclerotisations. After the anterior third of the segment, a distinct fold is apparent, subdividing the dorsal region of each segment into two distinct subregions (Figure 2B). Prominent setae arise from the trunk appendage elements. Femur and tibia are distally with a set of three to six setae each. Trunk segments with prominent long setae. These arise close to the posterior end of each segment and appear to form a kind of ring around the segment.
(11) Specimen 6647 (accession number nsw_epa_MANN16_batar_ck_220499) is well accessible (Figure 2C); it likely represents a penultimate larval stage. The specimen was collected during NSW EPA NSW EPA-MRHI survey, MANN-16, in the tributary of Batar Creek, New South Wales, Australia (31°40′57″ S, 152°40′7″ E), on 22 April 1999 by N. Waddell. Head with antennae, stylets, and labial palps. Antennae are elongated, subdivided into about six elements. Stylets are curved and tapering distally. Subdivision of the labial palps not well accessible. Trunk appendages with five major elements (coxa, trochanter, femur, tibia, tarsus), well preserved, but they are entangled with each other, partly obscuring subdivisions and details. Thorax is subdivided into pro-, meso-, and metathorax with a longer cervix anteriorly. Abdomen with 11 units. The cervix shows a longitudinal groove (Figure 2C). Trunk segments with prominent long setae. These arise close to the posterior end of each segment and appear to form a kind of ring around the segment.
(12) Specimen 6648 (accession number nsw_epa_MANN16_batar_ck_220499; in the same vial as specimen 6647) is well accessible (Figure 2D); it likely represents a final larval stage. The specimen was collected during the NSW EPA NSW EPA-MRHI survey, MANN-16, in the tributary of Batar Creek, New South Wales, Australia (31°40′57″ S, 152°40′7″ E), on 22 April 1999 by N. Waddell. Head with antennae, stylets, and labial palps. Antennae are elongated, subdivided into several elements. Stylets proximally straight, distally more curved, tapering distally. Subdivision of the labial palps not well accessible. Trunk appendages with five major elements (coxa, trochanter, femur, tibia, tarsus; Figure 2D) well preserved, but they are entangled with each other, partly obscuring subdivisions and details. Coxa is tubular and elongated; trochanter not well accessible; femur similar to coxa, but thinner; tibia similar to femur, but slightly shorter; tarsus quite slender again and tapering distally with a pair of claws. Thorax is subdivided into pro-, meso-, and metathorax with a longer cervix anteriorly. Abdomen with at least nine units. The cervix shows a longitudinal groove (Figure 2D). Trunk segments with prominent long setae. These arise close to the posterior end of each segment and appear to form a kind of ring around the segment.
(13) Specimen 6649 (accession number nsw_epa_MANN16_batar_ck_220499; in the same vial as specimens 6647, 6648) is well accessible (Figure 3A); it likely represents a penultimate or final larval stage. The specimen was collected during the NSW EPA NSW EPA-MRHI survey, MANN-16, in the tributary of Batar Creek, New South Wales, Australia (31°40′57″ S, 152°40′7″ E), on 22 April 1999 by N. Waddell. Head with antennae, stylets, and labial palps. Antennae are elongated, subdivided into several elements (about 12). Stylets are proximally straight, distally more curved, tapering distally. Labial palps are subdivided into at least four elements, proximal region not well accessible. Trunk appendages with five major elements (coxa, trochanter, femur, tibia, tarsus; Figure 3A), well preserved, but they are entangled with each other, partly obscuring subdivisions and details. Thorax is subdivided into pro-, meso-, and metathorax, with a long cervix anteriorly. Abdomen with at least 10 units. The cervix and prothorax show a longitudinal groove (Figure 2B). The trunk segments posterior to the prothorax with a distinct pattern of dorsal sclerotisations. After the anterior third of the segment, a distinct fold is apparent, subdividing the dorsal region of each segment into two distinct subregions (Figure 3A). Prominent setae arise from the trunk appendage elements. Femur and tibia are distally with a set of three to four setae each. Trunk segments with prominent long setae. These arise close to the posterior end of each segment and appear to form a kind of ring around the segment.

(14) Specimen 6650 (accession number nsw_epa_MANN16_batar_ck_220499; in the same vial as specimen 6647–6649) is well accessible (Figure 3B); it likely represents a final larval stage. The specimen was collected during the NSW EPA NSW EPA-MRHI survey, MANN-16, in the tributary of Batar Creek, New South Wales, Australia (31°40′57″S, 152°40′7″ E), on 22 April 1999 by N. Waddell. Head with antennae, stylets, and labial palps. Antennae are elongated, subdivided into several elements (about 14). Stylets are curved and tapering distally. Labial palps are subdivided into at least four elements apparent, proximal region not well accessible. Trunk appendages with five major elements (coxa, trochanter, femur, tibia, tarsus; Figure 3B), well preserved, but they are entangled with each other, partly obscuring subdivisions and details. Coxa is tubular and elongated; trochanter not accessible; femur similar to coxa, but shorter; tibia similar to femur, but a bit longer and wider; tarsus similar to tibia, but shorter and tapering distally with a pair of claws. Thorax is subdivided into pro-, meso-, and metathorax, with a long cervix anteriorly. Abdomen with at least 10 units. Prominent setae arise from the trunk appendage elements. Femur and tibia are distally with a set of four to six setae each. Few, less prominent setae also apparent further proximally, no clear pattern apparent. Trunk segments with prominent long setae. These arise close to the posterior end of each segment and appear to form a kind of ring around the segment.
(15) Specimen 6651 (accession number nsw_epa_BEGA23_Paddys_crk_171199) is well accessible (Figure 3C); it likely represents a penultimate or final larval stage. The specimen was collected during the NSW EPA-MRHI survey, BEGA-23, in Paddys Creek, New South Wales, Australia (36°33′3″ S, 149°80′8″ E), on 16 December 1999 by Jamie Potts. Head with elongate antennae, stylets, and one labial palp left. Antennae are subdivided into several elements (about 13). Stylets are curved and tapering distally. Labial palps are subdivided into five elements, the other one is broken at the second segment (Figure 3E). Trunk appendages with five major elements (coxa, trochanter, femur, tibia, tarsus) are mainly covered by the trunk. Coxa and trochanter are not accessible; femur tubular and elongated; tibia similar to femur, but thinner; tarsus quite slender again and tapering distally with a pair of claws. Thorax is subdivided into pro-, meso-, and metathorax with a long cervix anteriorly. Abdomen with at least 10 units. The cervix and prothorax show a longitudinal groove. The trunk segments posterior to the prothorax with a distinct pattern of dorsal sclerotisations. After the anterior third of the segment, a distinct fold is apparent, subdividing the dorsal region of each segment into two distinct subregions (Figure 3C). Prominent setae arise from the trunk appendage elements. Femur and tibia are distally with a set of three to five setae each. Trunk segments with prominent long setae. These arise close to the posterior end of each segment and appear to form a kind of ring around the segment.

### 3.3. Additional Fossil Larval Representatives of Nevrorthidae in Baltic Amber

**Figure 4:**
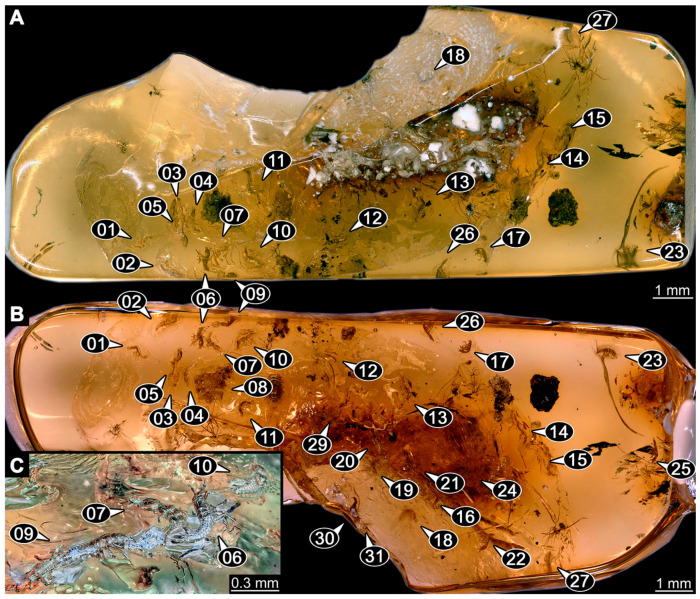
Nevrorthidae; fossil larvae preserved in Baltic amber; overview of large piece (PED 0871) with numerous specimens marked by numbers, ring light. (**A**). Side 1. (**B**). Side 2. (**C**). Close-up of some more tightly clustered specimens.

**Figure 5:**
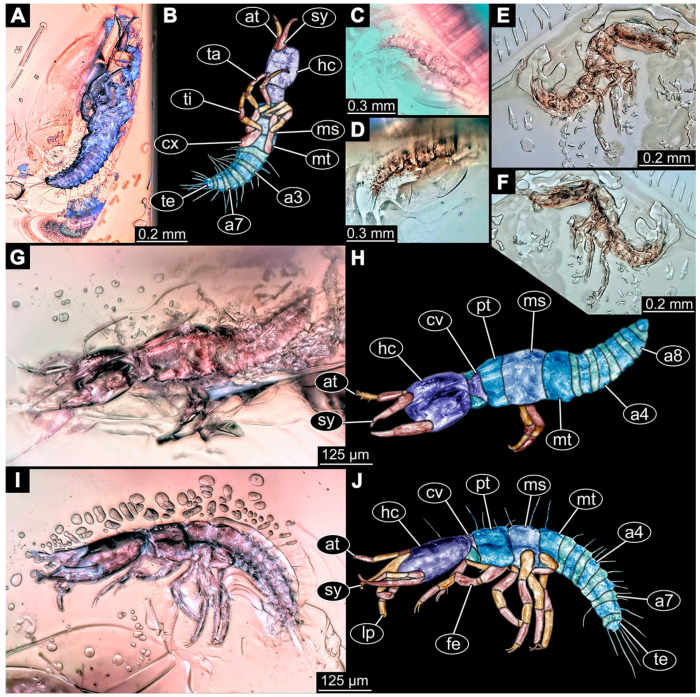
Nevrorthidae; fossil larvae preserved in Baltic amber; PED 0871, continued. (**A**–**D**). Specimen 6653 (specimen 2). (**A**). Ventral view. (**B**). Colour-marked version of (**A**). (**C**,**D**). Dorsal view. (**E**,**F**), Specimen 6652 (specimen 1). (**E**). Lateral view, right. (**F**). Lateral view, left. (**G**,**H**), Specimen 6654 (specimen 3). (**G**). Dorsal view. (**H**). Colour-marked version of (**G**). (**I**,**J**). Specimen 6655 (specimen 4). (**I**). Lateral view, left. (**J**). Colour-marked version of (**I**). (**A**,**C**,**G**,**I**). Cross-polarised light. (**D**–**F**). Ring light. Abbreviations: a1–7 = abdomen segments 1–7; at = antenna; cv = cervix (neck); cx = coxa; fe = femur; hc = head capsule; lp = labial palp; ms = mesothorax; mt = metathorax; pt = prothorax; sy = stylet; ta = tarsus; te = trunk end; ti = tibia.

**Figure 6:**
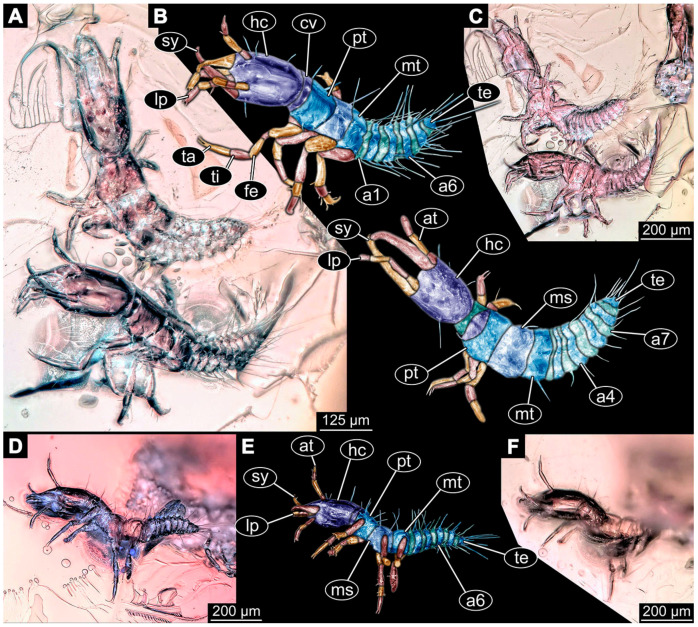
Nevrorthidae; fossil larvae preserved in Baltic amber; PED 0871, continued, cross-polarised light. (**A**–**C**). Specimen 6657 (specimen 6, lower one) and specimen 6658 (specimen 7, upper one). (**A**). Dorsal view. (**B**). Colour-marked version of (**A**). (**C**). Ventral view, image flipped. (**D**–**F**). Specimen 6656 (specimen 5). (**D**). Lateral view, right, image flipped. (**E**). Colour-marked version of (**D**). (**F**). Lateral view, left. Abbreviations: a1–7 = abdomen segments 1–7; at = antenna; cv = cervix (neck); fe = femur; hc = head capsule; lp = labial palp; ms = mesothorax; mt = metathorax; pt = prothorax; sy = stylet; ta = tarsus; te = trunk end; ti = tibia.

**Figure 7:**
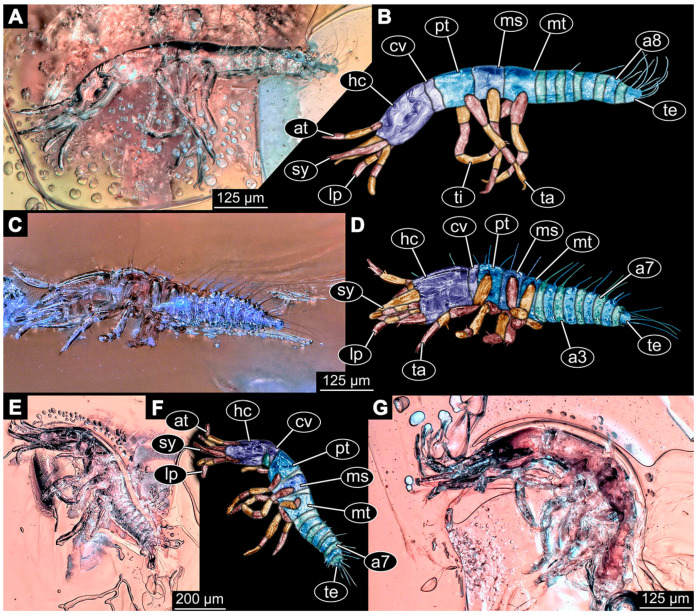
Nevrorthidae; fossil larvae preserved in Baltic amber; PED 0871, continued. (**A**,**B**). Specimen 6659 (specimen 8). (**A**). Lateral view, left. (**B**). Colour-marked version of (**A**). (**C**,**D**), Specimen 6660 (specimen 9). (**C**). Ventral view. (**D**). Colour-marked version of (**C**). (**E**,**F**). Specimen 6661 (specimen 10). (**E**). Lateral view, right, image flipped. (**F**). Colour-marked version of (**E**). (**G**). Specimen 6662 (specimen 11), lateral view, left. (**A**,**E**,**G**). Cross-polarised light. (**C**). Ring light. Abbreviations: a1–8 = abdomen segments 1–8; at = antenna; cv = cervix (neck); hc = head capsule; lp = labial palp; ms = mesothorax; mt = metathorax; pt = prothorax; sy = stylet; ta = tarsus; te = trunk end; ti = tibia.

**Figure 8:**
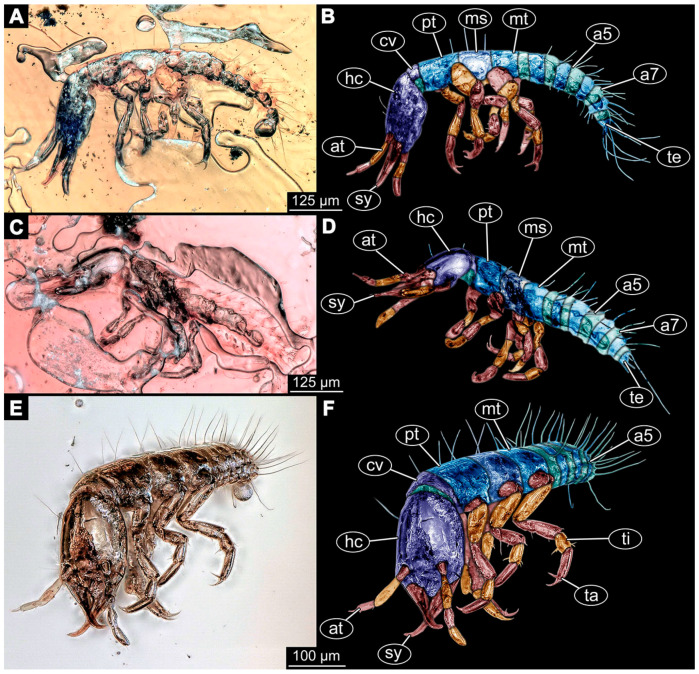
Nevrorthidae; fossil larvae preserved in Baltic amber; PED 0871, continued, images flipped. (**A**,**B**). Specimen 6663 (specimen 12). (**A**). Lateral view, right. (**B**). Colour-marked version of (**A**). (**C**,**D**). Specimen 6664 (specimen 13). (**C**). Lateral view, right. (**D**). Colour-marked version of (**C**). (**E**,**F**), Specimen 6668 (specimen 17). (**E**). Lateral view, right. (**F**). Colour-marked version of (**E**). (**A**,**C**). Cross-polarised light. (**E**). Ring light. Abbreviations: a1–7 = abdomen segments 1–7; at = antenna; cv = cervix (neck); hc = head capsule; lp = labial palp; ms = mesothorax; mt = metathorax; pt = prothorax; sy = stylet; ta = tarsus; te = trunk end; ti = tibia.

**Figure 9:**
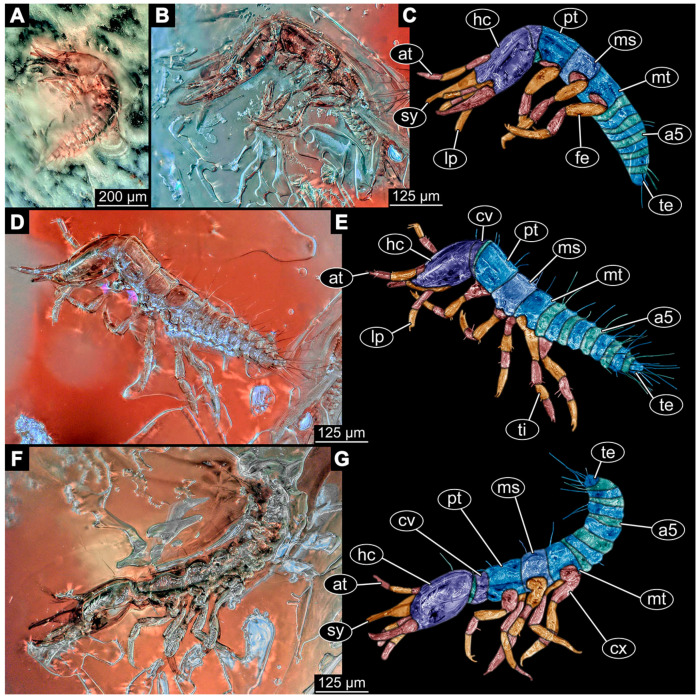
Nevrorthidae; fossil larvae preserved in Baltic amber; PED 0871, continued. (**A**). Specimen 6669 (specimen 18). (**A**). Lateral view, right, image flipped. (**B**,**C**). Specimen 6670 (specimen 19). (**B**). Lateral view, left. (**C**). Colour-marked version of (**B**). (**D**,**E**). Specimen 6671 (specimen 20). (**D**). Lateral view, right, image flipped. (**E**). Colour-marked version of (**D**). (**F**,**G**). Specimen 6672 (specimen 21). (**F**). Lateral view, left. (**G**). Colour-marked version of (**F**). (**A**). Cross-polarised light. (**B**,**D**,**F**). Ring light. Abbreviations: a1–5 = abdomen segments 1–5; at = antenna; cv = cervix (neck); cx = coxa; fe = femur; hc = head capsule; lp = labial palp; ms = mesothorax; mt = metathorax; pt = prothorax; sy = stylet; te = trunk end; ti = tibia.

**Figure 10:**
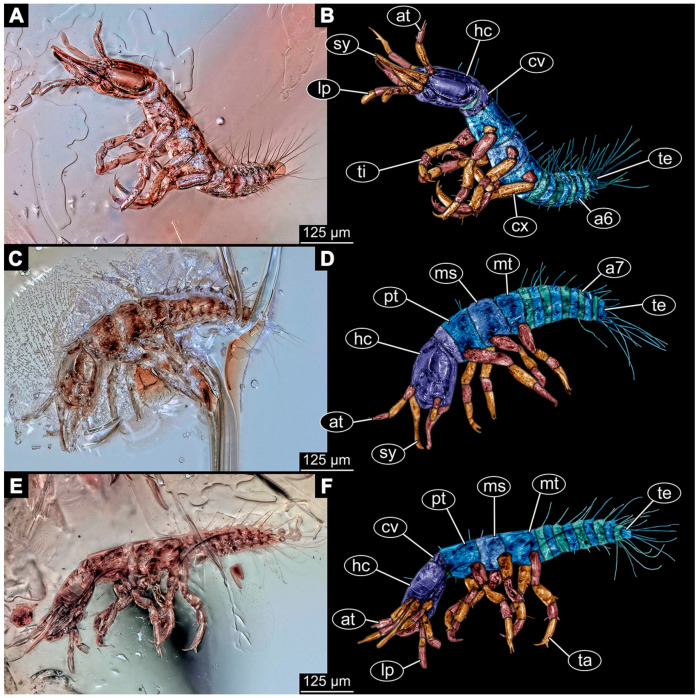
Nevrorthidae; fossil larvae preserved in Baltic amber; PED 0871, continued, ring light, images flipped, lateral view, right. (**A**,**B**). Specimen 6673 (specimen 22). (**A**). Overview. (**B**). Colour-marked version of (**A**). (**C**,**D**). Specimen 6674 (specimen 23). (**C**). Overview. (**D**). Colour-marked version of (**C**). (**E**,**F**). Specimen 6676 (specimen 25). (**E**). Overview. (**F**). Colour-marked version of (**E**). Abbreviations: a6–7 = abdomen segments 6–7; at = antenna; cv = cervix (neck); cx = coxa; hc = head capsule; lp = labial palp; ms = mesothorax; mt = metathorax; pt = prothorax; sy = stylet; ta = tarsus; te = trunk end; ti = tibia.

**Figure 11:**
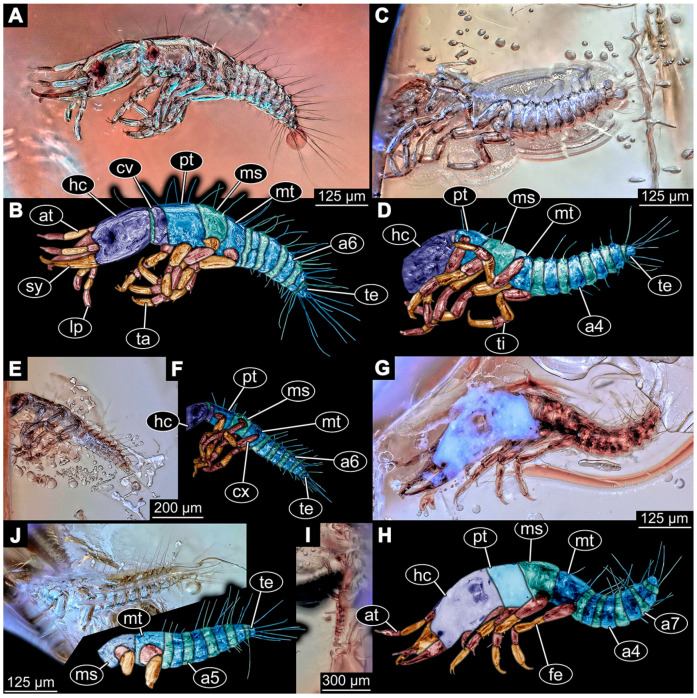
Nevrorthidae; fossil larvae preserved in Baltic amber; PED 0871, continued, ring light. (**A**,**B**). Specimen 6680 (specimen 29). (**A**). Lateral view, right, image flipped. (**B**). Colour-marked version of (**A**). (**C**,**D**). Specimen 6678 (specimen 27). (**C**). Lateral view, left. (**D**). Colour-marked version of (**C**). (**E**,**F**). Specimen 6677 (specimen 26). (**E**). Lateral view, left. (**F**). Colour-marked version of (**E**). (**G**,**H**). Specimen 6681 (specimen 30). (**G**). Lateral view, left. (**H**). Colour-marked version of (**G**). (**I**). Specimen 6679 (specimen 28), dorsal view. (**J**). Specimen 6682 (specimen 31), lateral view, left (upper) and colour-marked version (lower). Abbreviations: a4–7 = abdomen segments 4–7; at = antenna; cv = cervix (neck); cx = coxa; fe = femur; hc = head capsule; lp = labial palp; ms = mesothorax; mt = metathorax; pt = prothorax; sy = stylet; ta = tarsus; te = trunk end; ti = tibia.

**Figure 12:**
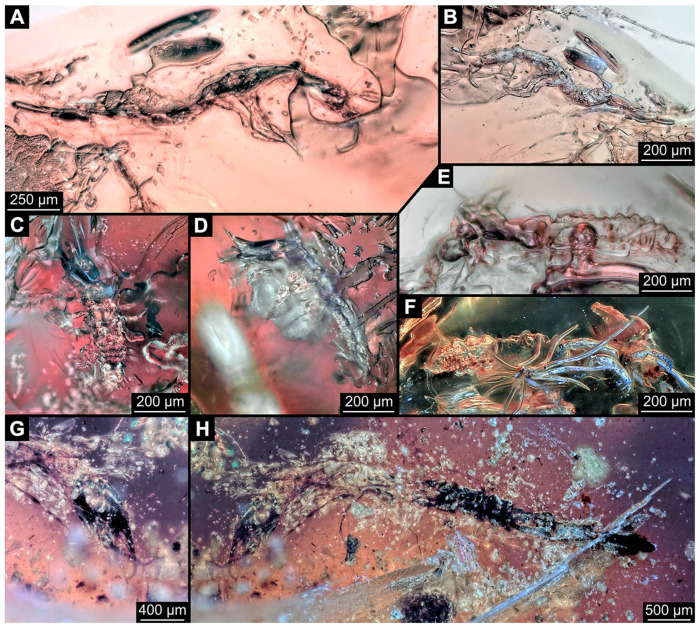
Nevrorthidae; fossil larvae preserved in Baltic amber and Kachin amber. (**A**–**F**). PED 0871, continued, Baltic amber. (**A**,**B**). Specimen 6665 (specimen 14). (**A**). Lateral view, right. (**B**). Lateral view, left. (**C**). Specimen 6667 (specimen 16), dorsal view. (**D**). Specimen 6675 (specimen 24), lateral view, left. (**E**,**F**). Specimen 6666 (specimen 15). (**E**). Lateral view, left. (**F**). Lateral view, right. (**G**,**H**). Specimen 6717 (PED 2996), Kachin amber. (**G**). Close-up of head with crossed stylets, dorsal view. (**H**). Lateral view, right. (**A**,**C**–**E**). Cross-polarised light. (**B**,**F**–**H**). Ring light.

**Figure 13:**
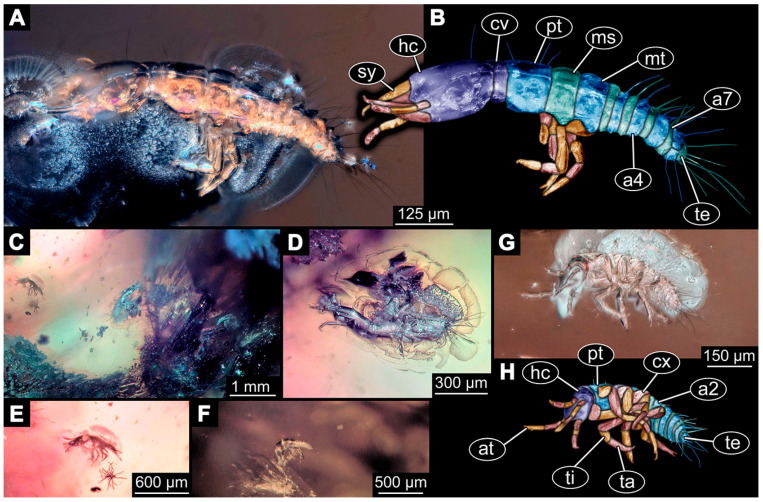
Nevrorthidae; fossil larvae preserved in Baltic amber (PED 1373). (**A**,**B**). Specimen 6683 (specimen 1). (**A**). Lateral view, right, image flipped. (**B**). Colour-marked version of (**A**). (**C**). Overview of specimens 2–4 (2 left, 3 right, 4 middle). (**D**). Specimen 6686 (specimen 4), lateral view, right, image flipped. (**E**). Specimen 6684 (specimen 2), lateral view, left. (**F**). Specimen 6685 (specimen 3), lateral view, right, image flipped. (**G**,**H**). Specimen 6687 (specimen 5). (**G**). Ventral view. (**H**). Colour-marked version of (**G**). (**A**,**C**–**F**). Cross-polarised light. (**G**). Ring light. Abbreviations: a2–7 = abdomen segments 2–7; at = antenna; cv = cervix (neck); cx = coxa; hc = head capsule; ms = mesothorax; mt = metathorax; pt = prothorax; sy = stylet; ta = tarsus; te = trunk end; ti = tibia.

**Figure 14:**
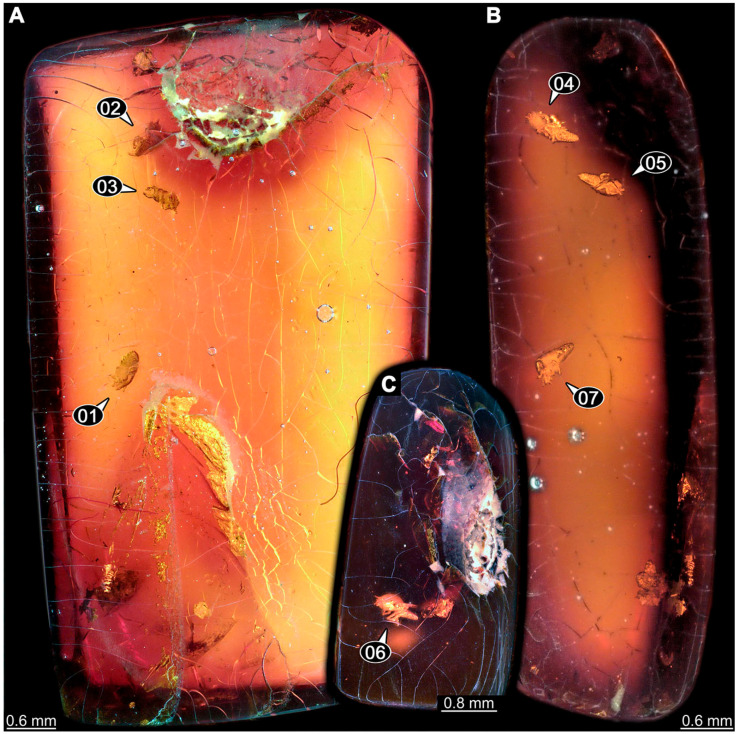
Nevrorthidae; fossil larvae preserved in Baltic amber; overview of larger piece (NHMD 115214) with several specimens marked by numbers, ring light. (**A**). Side 1 (“top”). (**B**). Side 2 (“side”). (**C**). Side 3 (“front”).

**Figure 15:**
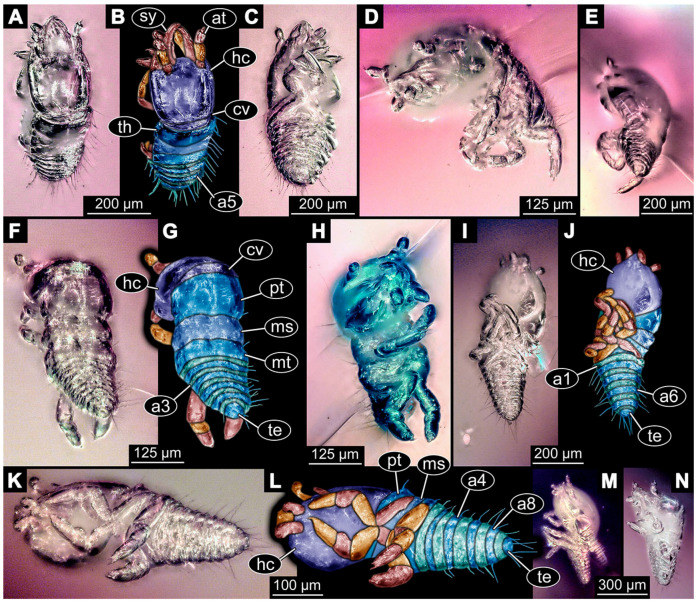
Nevrorthidae; fossil larvae preserved in Baltic amber; NHMD 115214, continued. (**A**–**C**). Specimen 6688 (specimen 1). (**A**). Dorsal view. (**B**). Colour-marked version of (**A**). (**C**). Ventral view. (**D**,**E**). Specimen 6690 (specimen 3). (**D**). Lateral view, right, image flipped. (**E**). Ventral view. (**F**–**H**). Specimen 6689 (specimen 2). (**F**). Dorsal view. (**G**). Colour-marked version of (**F**). (**H**). Ventral view. (**I**,**J**). Specimen 6691 (specimen 4). (**I**). Ventral view. (**J**). Colour-marked version of (**I**). (**K**,**L**). Specimen 6692 (specimen 5). (**K**). Ventral view. (**L**). Colour-marked version of (**K**). (**M**). Specimen 6694 (specimen 7), ventral view. (**N**). Specimen 6693 (specimen 6), ventral view. (**A**,**C**,**D**,**F**,**I**,**K**,**N**). Ring light. (**E**,**M**). Mixed light. (**H**). Cross-polarised light. Abbreviations: a1–8 = abdomen segments 1–8; at = antenna; cv = cervix (neck); hc = head capsule; ms = mesothorax; mt = metathorax; pt = prothorax; sy = stylet; te = trunk end; th = thorax.

**Figure 16:**
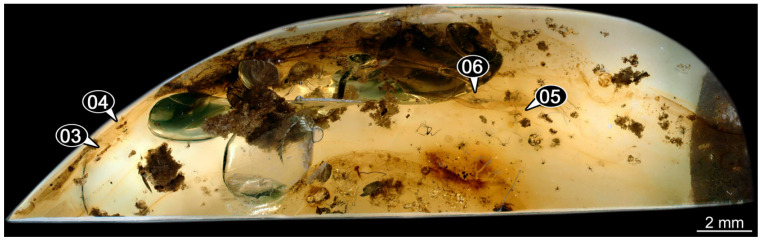
Nevrorthidae; fossil larvae preserved in Baltic amber; overview of piece (CCHH 1124-4b) with several specimens marked by numbers, ring light.

**Figure 17:**
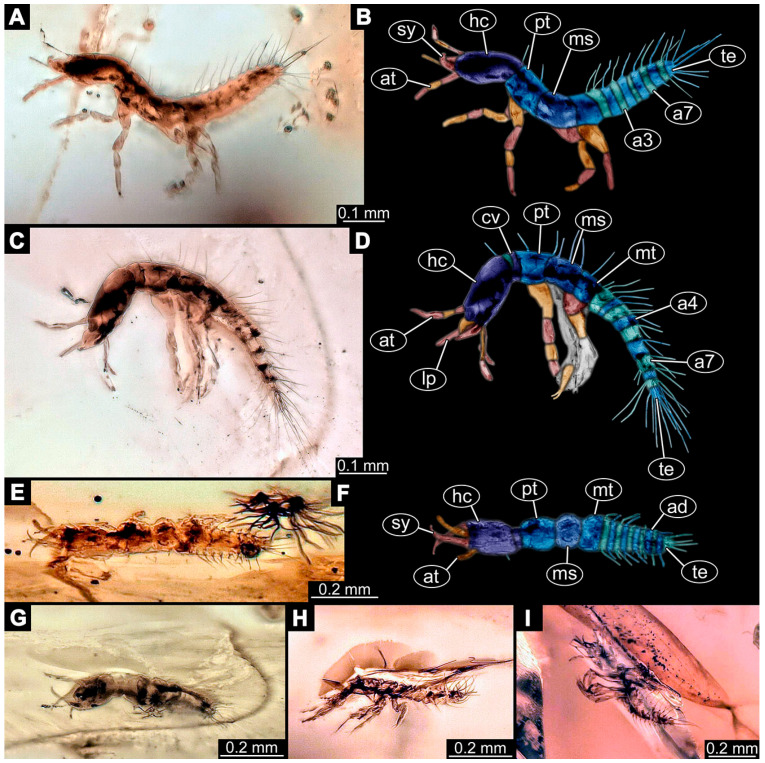
Nevrorthidae; fossil larvae preserved in Baltic amber; CCHH 1124-4b, continued. (**A**,**B**). Specimen 6695 (specimen 1), images flipped. (**A**). Lateral view, right. (**B**). Colour-marked version of (**A**). (**C**,**D**). Specimen 6696 (specimen 2). (**C**). Lateral view, left. (**D**). Colour-marked image of (**C**). (**E**,**F**). Specimen 6697 (specimen 3). (**E**). Dorsal view. (**F**). Colour-marked version of (**E**). (**G**). Specimen 6698 (specimen 4), dorsal view. (**H**). Specimen 6699 (specimen 5), lateral view, right, image flipped. (**I**). Specimen 6700 (specimen 6), lateral view, right. image flipped. (**A**,**E**,**G**–**I**). Ring light. (**C**). Cross-polarised light. Abbreviations: a3–7 = abdomen segments 3–7; ad = abdomen; at = antenna; cv = cervix (neck); hc = head capsule; lp = labial palp; ms = mesothorax; mt = metathorax; pt = prothorax; sy = stylet; te = trunk end.

**Figure 18:**
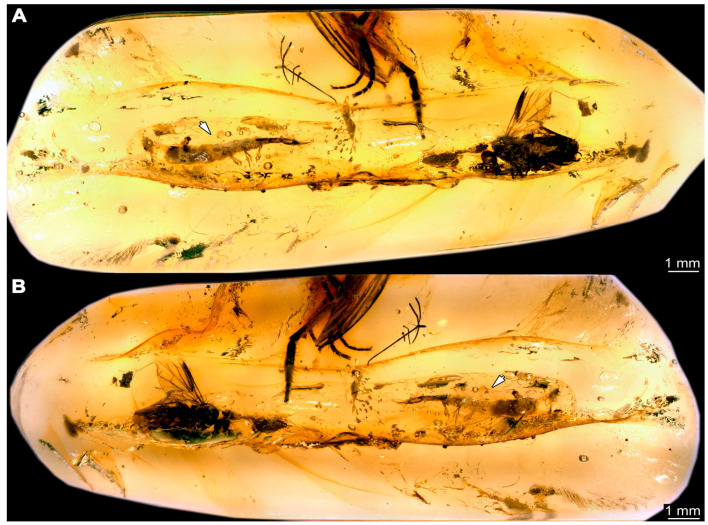
Nevrorthidae; fossil larvae preserved in Baltic amber; overview of piece (CCHH 1270-4) with two specimens (one apparent here, see arrows), ring light. (**A**). Side 1. (**B**). Side 2.

**Figure 19:**
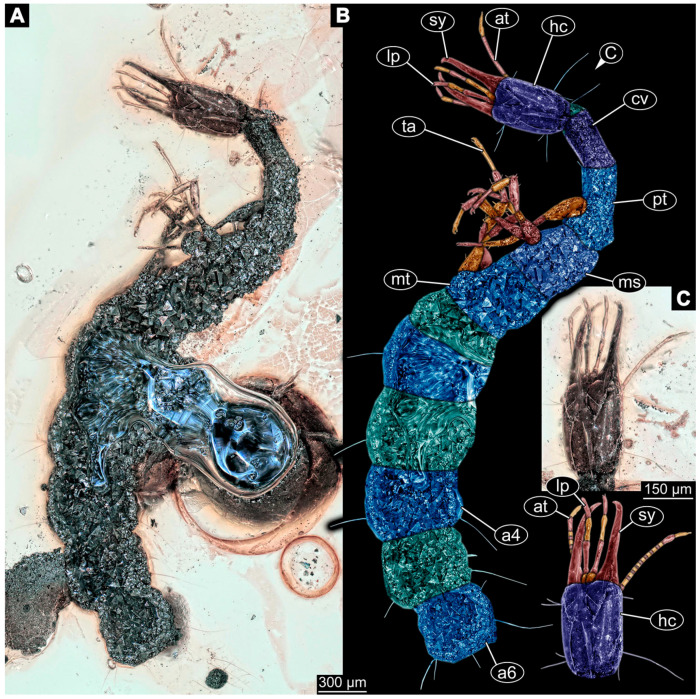
Nevrorthidae; fossil larvae preserved in Baltic amber; CCHH 1270-4, continued; specimen 6701 (specimen 1), ring light. (**A**). Lateral view, left. (**B**). Colour-marked version of (**A**). (**C**). Close-up of head in dorsal view (upper) and colour-marked version (lower). Abbreviations: a4–6 = abdomen segments 4–6; at = antenna; cv = cervix (neck); hc = head capsule; lp = labial palp; ms = mesothorax; mt = metathorax; pt = prothorax; sy = stylet; ta = tarsus; te = trunk end.

**Figure 20:**
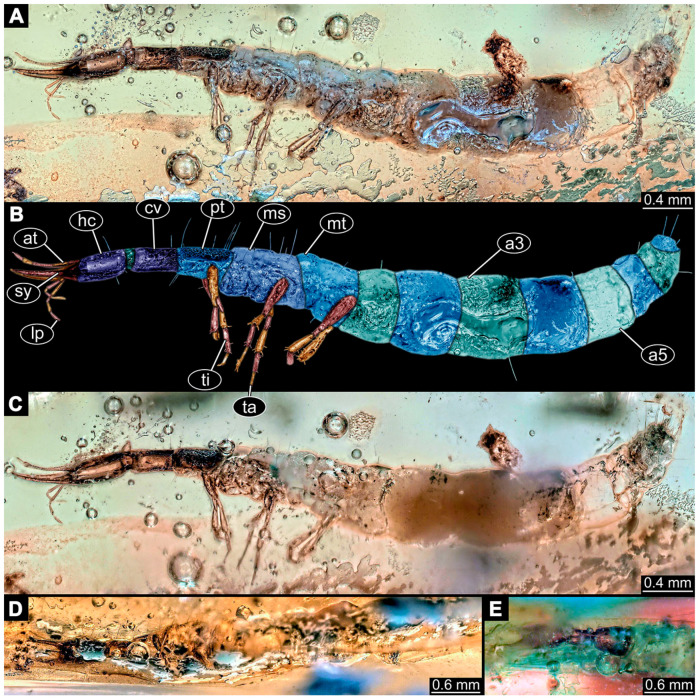
Nevrorthidae; fossil larvae preserved in Baltic amber; CCHH 1270-4, continued; specimen 6702 (specimen 2). (**A**). Lateral view, right, image flipped. (**B**). Colour-marked version of (**A**). (**C**). Lateral view, left. (**D**). Ventral view. (**E**). Dorsal view. (**A**,**C**,**D**). Ring light. (**E**). Cross-polarised light. Abbreviations: a3–5 = abdomen segments 3–5; at = antenna; cv = cervix (neck); hc = head capsule; lp = labial palp; ms = mesothorax; mt = metathorax; pt = prothorax; sy = stylet; ta = tarsus; ti = tibia.

(16) Specimen 6652 (PED 0871 spec 1) is preserved together with numerous specimens in a single piece of Baltic amber (Figure 4). The specimen is accessible in lateral view from both sides of the body (Figure 5E,F). Other aspects are not accessible. The specimen shows the overall slender morphology known for older larvae of Nevrorthidae in the anterior region, but especially the posterior trunk appears much less slender, the segments of the abdomen are extremely short. The details of the head (antennae, stylets, and labial palps) are not well accessible. Trunk appendages with five major elements (coxa, trochanter, femur, tibia, tarsus), but subdivisions not well visible. Subdivisions of the thorax and abdomen largely obscured by bubbles. A few elongate setae arise from the thorax and abdomen segments.

(17) Specimen 6653 (PED 0871 spec 2) is accessible from the ventral side and less well from the dorsal side (Figure 5A–D). It is preserved in the same amber piece as specimen 6652. Well-accessible details include the abdomen segments with several setae and parts of the thorax appendages. Other aspects are not well accessible. The specimen shows the overall slender morphology known for older larvae of Nevrorthidae in the anterior region, but especially the posterior trunk appears much less slender, the segments of the abdomen are extremely short. Most of the anterior body is destroyed. Of the head appendages, only one stylet and a part of the antenna remain. Stylets are proximally straight, then they get more curved, tapering distally. Parts of trunk appendages with five major elements (coxa, trochanter, femur, tibia, tarsus; Figure 5B). Coxa is tubular and bellied; trochanter not visible; femur a bit thinner and elongated; tibia shorter and thicker than the femur; tarsus a bit longer than tibia and tapering distally. Also, the ten abdomen units with their subdivisions are well accessible. Numerous elongate setae arise from the abdomen segments; insertion areas obscured. They seem to arise in the middle of each segment.
(18) Specimen 6654 (PED 0871 spec 3) is only accessible in dorsal view (Figure 5G,H). Other aspects are not accessible. It is preserved in the same amber piece as specimens 6652 and 6653. The specimen shows the overall slender morphology known for older larvae of Nevrorthidae in the anterior region, but especially the posterior trunk appears much less slender, the segments of the abdomen are extremely short. Head outline well apparent in dorsal view with a short cervix. Head with stylets and one stout antenna left. The rest of the head appendages are not accessible. Stylets taper distally; they are straight proximally broader, but sharply curved more distally. The antenna, subdivided into three elements, appears a bit thicker than at other specimens (Figure 5H). Most of the subdivisions of the thorax and trunk appendages are obscured. On the trunk no setae are visible.
(19) Specimen 6655 (PED 0871 spec 4) is only accessible from the lateral left side (Figure 5I,J), other aspects are not accessible. It is preserved in the same amber piece as specimens 6652–6654. The specimen shows the overall slender morphology known for older larvae of Nevrorthidae in the anterior region, but especially the posterior trunk appears much less slender, the segments of the abdomen are extremely short. The amber is rather clear and the overall outline is easily accessible. The specimen is a bit curved dorsally. The head outline with a short cervix is well apparent. Head with antennae, stylets, and labial palps. Subdivisions are a bit obscured by Verlumung. Antennae elongate slender, subdivided into three elements. The stylets are curved and tapering distally. They seem to be as long as the antennae. Labial palps are present, but mostly covered by the stylets. Trunk appendages with five major elements (coxa, trochanter, femur, tibia, tarsus; Figure 5J), well preserved, but they are entangled with each other, partly obscuring subdivisions and details. Coxa is tubular and elongate; trochanter significantly shorter; femur shorter than the coxa and a bit thicker; tibia thinner and a bit shorter again; tarsus quite slender, elongate and longer than tibia, distally with a pair of claws. The thorax is clearly subdivided into pro-, meso-, and metathorax. Body carrying numerous elongate setae; insertion areas obscured. They seem to be longer at the trunk end.
(20) Specimen 6656 (PED 0871 spec 5) is accessible in left and right lateral view of the body (Figure 6D–F). The distal body parts of the left view are obscured, other aspects are not accessible. The specimen is preserved in the same amber piece as specimens 6652–6655. It shows the overall slender morphology known for older larvae of Nevrorthidae in the anterior region, but especially the posterior trunk appears much less slender, the segments of the abdomen are extremely short. Furthermore, it seems a bit smaller than the other specimens. Overall outline is well accessible from the lateral right side of the body. The head with a short cervix is seen in ventral view with antennae, stylets, and labial palps. Antennae elongate slender, subdivided into three elements (Figure 6F) with a few setae. The stylets are curved and tapering distally. Labial palps are slightly shorter than antennae, subdivided into at least three elements apparent, proximal region not accessible. Trunk appendages with five major elements (coxa, trochanter, femur, tibia, tarsus; Figure 6E). Coxa is tubular and elongated; trochanter not accessible; femur tubular and elongated as well; tibia shorter and thicker than femur; tarsus quite slender, elongate and longer than tibia, distally with a pair of claws. The thorax is clearly subdivided into pro-, meso-, and metathorax. Body carrying numerous elongate setae; most insertion areas are obscured. They seem to be longer at the trunk end.

(21) Specimen 6657 (PED 0871 spec 6) is accessible in dorsal and ventral view (Figure 6A lower, B upper, C lower). It is preserved in the same amber piece as specimens 6652–6656. The specimen shows the overall slender morphology known for older larvae of Nevrorthidae in the anterior region, but especially the posterior trunk appears much less slender, the segments of the abdomen are extremely short. Overall outline is well accessible from both sides. Head with stout antennae, stylets, and labial palps. Antennae are a bit thicker in shape than in other specimens and subdivided into three elements (Figure 6B, upper). The stylets are curved with a broad proximal region and tapering distally. Labial palps are slightly longer than antennae, subdivided into at least three elements apparent, proximal region not accessible. Trunk appendages with five major elements (coxa, trochanter, femur, tibia, tarsus; Figure 6B, upper). Coxa is tubular and elongated; trochanter conical and shorter; femur tubular, shorter than coxa; tibia similar, but shorter; tarsus similar as well, but tapering distally and with a pair of claws. The thorax is clearly subdivided into pro-, meso-, and metathorax with a short cervix and the abdomen is divided into 10 units. All over the body, several setae arise. Most insertion areas are obscured by Verlumung, but at the abdomen, they seem to arise in the middle of each segment.
(22) Specimen 6658 (PED 0871 spec 7) is accessible in dorsal and ventral view (Figure 6A upper B lower, C upper). It is preserved in the same amber piece as specimens 6652–6657. The specimen shows the overall slender morphology known for older larvae of Nevrorthidae in the anterior region, but especially the posterior trunk appears much less slender, the segments of the abdomen are extremely short. Overall outline is well accessible from both sides. The subdivisions of the head appendages (antennae, stylets, and labial palps) are largely obscured by Verlumung. Only one stout antenna, both stylets, and one labial palp remain. Antenna is elongated and subdivided into three elements. The stylets taper distally; they are straight and broad proximally, but sharply curved more distally. Labial palp is elongated and slightly longer than antenna, subdivided into four elements. Trunk appendages with five major elements (coxa, trochanter, femur, tibia, tarsus), but proximal not well visible; distally with a pair of claws (Figure 6B, lower). The thorax is subdivided into pro-, meso-, and metathorax. Along the short cervix and thorax, a distinct fold is apparent. Abdomen slightly obscured by Verlumung, but the ten abdomen units still well accessible. A few setae arise from the body, most of them at the abdomen segments. They seem to arise in the middle of each segment.
(23) Specimen 6659 (PED 0871 spec 8) is only accessible from left lateral side of the body (Figure 7A,B). It is preserved in the same amber piece as specimens 6652–6658. The specimen shows the overall slender morphology known for older larvae of Nevrorthidae in the anterior region, but especially the posterior trunk appears much less slender, the segments of the abdomen are extremely short. Overall outline is a bit obscured by tiny bubbles, but still accessible. Head with one antenna, both stylets, and labial palps. Antenna is elongated, quite slender, and tapering distally, subdivided into three elements. Stylets are elongate and tapering distally, but mostly cover each other. Labial palps are slightly shorter than antenna, subdivided into at least three elements apparent, proximal region not accessible. Trunk appendages with five major elements (coxa, trochanter, femur, tibia, tarsus; Figure 7B), well preserved, but they are entangled with each other, partly obscuring subdivisions and details. Coxa is tubular and elongated; trochanter not accessible; femur tubular and slightly shorter than coxa; tibia similar; tarsus quite slender, elongate and distally with a pair of claws. The thorax is clearly subdivided into pro-, meso-, and metathorax with a short cervix anteriorly. Only at the trunk end are a few elongate setae visible. The insertion areas are obscured.

(24) Specimen 6660 (PED 0871 spec 9) is only accessible from ventral view of the body (Figure 7C,D). It is preserved in the same amber piece as specimens 6652–6659. The specimen shows the overall slender morphology known for older larvae of Nevrorthidae in the anterior region, but especially the posterior trunk appears much less slender, the segments of the abdomen are extremely short. The overall outline is well accessible. The head appendages (antennae, stylets, labial palps) and the abdomen units are a bit obscured by Verlumung. Stout antennae, with a few setae elongate slender; subdivision into at least three elements. Stylets with a broad proximal region tapering distally. The distal parts of the stylets are mostly covered by the labial palps, which are slightly shorter than the antennae and subdivided into four elements. Trunk appendages with five major elements (coxa, trochanter, femur, tibia, tarsus; Figure 7D). Only one leg is fully accessible. Coxa tubular, elongate; trochanter not accessible; femur tubular and similar to the coxa; tibia similar, but shorter than femur; tarsus similar, but longer than tibia, distally with a pair of claws. The thorax is clearly subdivided into pro-, meso-, and metathorax with a short cervix anteriorly. In spite of Verlumung the subdivisions of the abdomen are well accessible. The ten abdomen units bear several setae, which seem to have their insertion areas at the middle of each unit.
(25) Specimen 6661 (PED 0871 spec 10) is only accessible from the right lateral side of the body (Figure 7E,F). It is preserved in the same amber piece as specimens 6652–6660. The specimen shows the overall slender morphology known for older larvae of Nevrorthidae in the anterior region, but especially the posterior trunk appears much less slender, the segments of the abdomen are extremely short. Despite the Verlumung, the overall outline is well accessible. Head with antennae, stylets, and labial palps. Stout antennae subdivided into three elements. The stylets taper distally. They are straight proximally with a broad proximal region, but a bit curved more distally. Labial palps look similar to the antennae, subdivided into at least three elements apparent, proximal region not accessible. Trunk appendages with five major elements (coxa, trochanter, femur, tibia, tarsus; Figure 7D), well preserved, but they are entangled with each other, partly obscuring subdivisions and details. The thorax is clearly subdivided into pro-, meso-, and metathorax with a short cervix anteriorly. Only a few setae arise at the ten abdomen units. The insertion areas are mostly obscured by Verlumung.
(26) Specimen 6662 (PED 0871 spec 11) is only accessible from the left lateral side of the body (Figure 7G). It is preserved in the same amber piece as specimens 6652–6661. The specimen shows the overall slender morphology known for older larvae of Nevrorthidae in the anterior region, but especially the posterior trunk appears much less slender, the segments of the abdomen are extremely short. The specimen is strongly curved ventrally, basically adopting a U-shaped posture. Overall body outline well accessible, yet most details, especially of the more distal body and the head are obscured by Verlumung, dark spots, and bubbles. Head with antennae, stylets, and labial palps, but details such as subdivision obscured. Trunk appendages with five major elements (coxa, trochanter, femur, tibia, tarsus), well preserved, but they are entangled with each other, partly obscuring subdivisions and details. No setae or clear subdivision of the trunk are accessible.
(27) Specimen 6663 (PED 0871 spec 12) is only accessible from the lateral right side of the body (Figure 8A,B). It is preserved in the same amber piece as specimens 6652–6662. The specimen shows the overall slender morphology known for older larvae of Nevrorthidae in the anterior region, but especially the posterior trunk appears much less slender, the segments of the abdomen are extremely short. The specimen is a bit curved ventrally, basically adopting a U-shaped posture. Overall body outline well accessible. Head with stout antennae and stylets. Antennae are elongated and subdivided into three elements, tapering distally. The labial palps are totally covered by the head and not accessible. Trunk appendages with five major elements (coxa, trochanter, femur, tibia, tarsus; Figure 8B), well preserved, but they are entangled with each other, partly obscuring subdivisions and details. Most accessible are pairs of claws, which are located at the distal end of the tarsi. The thorax is clearly subdivided into pro-, meso-, and metathorax with a short cervix anteriorly. Numerous elongate setae arise from the ten abdomen units, they seem to be longer at the trunk end.

(28) Specimen 6664 (PED 0871 spec 13) is only accessible from the lateral right side of the body (Figure 8C,D). It is preserved in the same amber piece as specimens 6652–6663. The specimen shows the overall slender morphology known for older larvae of Nevrorthidae in the anterior region, but especially the posterior trunk appears much less slender, the segments of the abdomen are extremely short. The overall outline is more difficult to identify. Large parts of the body are obscured by bubbles. Head with very stout antennae, stylets, and labial palps. Antennae are elongated and tapering distally. Stylets are straight with a broad proximal region and tapering distally as well. The labial palps are mostly not accessible. Trunk appendages with five major elements (coxa, trochanter, femur, tibia, tarsus; Figure 8D), well preserved, but they are entangled with each other, partly obscuring subdivisions and details. The thorax is subdivided into pro-, meso-, and metathorax with a short cervix anteriorly. Numerous elongate setae arise from the ten abdomen units.
(29) Specimen 6668 (PED 0871 spec 17) is only accessible from the lateral right side of the body (Figure 8E,F). It is preserved in the same amber piece as specimens 6652–6664. The specimen shows the overall slender morphology known for older larvae of Nevrorthidae in the anterior region, but especially the posterior trunk appears much less slender, the segments of the abdomen are extremely short. At this part the amber is very clear, hence the overall body outline of the specimen is well accessible. Head with antennae, stylets, and labial palps. Stout antennae are elongated and show subdivision into three to four elements. Stylets taper distally. They are straight proximally, but sharply curved more distally. The labial palps are covered by the head and not accessible. Trunk appendages with five major elements (coxa, trochanter, femur, tibia, tarsus; Figure 8F). Coxa appears conical; trochanter tubular, elongate; femur similar, but shorter; tibia similar as well, but shorter than femur; tarsus similar too, but taper distally with a pair of claws at the end. The thorax is subdivided into pro-, meso-, and metathorax with a short cervix anteriorly. Prominent setae arise from the trunk appendage elements. Femur and tibia are distally with a set of three setae each. Few, less prominent setae also apparent further proximally, no clear pattern apparent. Trunk segments with prominent long setae. These arise in the middle of each segment. The abdomen is subdivided into at least six units apparent, posterior region not accessible.
(30) Specimen 6669 (PED 0871 spec 18) is only accessible from the lateral right side of the body (Figure 9A). It is preserved in the same amber piece as specimens 6652–6664 and 6668. The specimen shows the overall slender morphology known for older larvae of Nevrorthidae in the anterior region, but especially the posterior trunk appears much less slender, the segments of the abdomen are extremely short. The specimen is a bit curved ventrally, basically adopting a U-shaped posture. The overall body outline is partly difficult to identify. Head with antennae, stylets, and labial palps, but the details, such as subdivision are obscured. Trunk appendages are strongly verlumt, only vague outlines become apparent. Despite the Verlumung, the thorax shows a subdivision into pro-, meso-, and metathorax. A few setae arise from the trunk, yet due to the Verlumung, their insertion areas are obscured.

(31) Specimen 6670 (PED 0871 spec 19) is only accessible from the lateral left side of the body (Figure 9B,C). It is preserved in the same amber piece as specimens 6652–6664, 6668, and 6669. The specimen shows the overall slender morphology known for older larvae of Nevrorthidae in the anterior region, but especially the posterior trunk appears much less slender, the segments of the abdomen are extremely short. The outline of the specimen is accessible. Head with stout antennae, stylets, and labial palps. Antennae elongate and subdivide into three elements. Stylets taper distally. They are straight proximally with a broad proximal region, but curved more distally. The labial palps are mostly covered by the head, subdivided into at least three elements apparent, proximal region not accessible. Trunk appendages with five major elements (coxa, trochanter, femur, tibia, tarsus; Figure 9C), well preserved, but they are entangled with each other, partly obscuring subdivisions and details. The thorax is subdivided into pro-, meso-, and metathorax with a short cervix anteriorly. Only a few setae arise from the ten abdomen units.
(32) Specimen 6671 (PED 0871 spec 20) is only accessible from the lateral right side of the body (Figure 9D,E). It is preserved in the same amber piece as specimens 6652–6664 and 6668–6670. The specimen shows the overall slender morphology known for older larvae of Nevrorthidae in the anterior region, but especially the posterior trunk appears much less slender, the segments of the abdomen are extremely short. The overall body outline is clearly accessible. Head with antennae, stylets, and labial palps. Stout antennae taper distally with a few setae. The stylets are mostly covered in this position by the head capsule, as well as the labial palps. Labial palps are subdivided into at least two elements apparent, proximal region not accessible. Trunk appendages with five major elements (coxa, trochanter, femur, tibia, tarsus; Figure 9E). The thorax is subdivided into pro-, meso-, and metathorax with a short cervix anteriorly. Numerous elongate setae arise from the trunk.
(33) Specimen 6672 (PED 0871 spec 21) is only accessible from the lateral left side of the body (Figure 9F,G). It is preserved in the same amber piece as specimens 6652–6664 and 6668–6671. The specimen shows the overall slender morphology known for older larvae of Nevrorthidae in the anterior region, but especially the posterior trunk appears much less slender, the segments of the abdomen are extremely short. The overall body outline is well accessible. Head with one antenna (left), stylets, and labial palps. Antennae elongate and subdivided into three elements. Stylets taper distally. They are straight proximally with a broad proximal region, but curved more distally. The labial palps are mostly covered by the stylets, subdivided into at least three elements apparent, proximal region not accessible. Trunk appendages with five major elements (coxa, trochanter, femur, tibia, tarsus; Figure 9G), well preserved, but they are entangled with each other, partly obscuring subdivisions and details. Most accessible are the pairs of claws, which are located at the distal end of the tarsi. The thorax is clearly subdivided into pro-, meso-, and metathorax with a short cervix anteriorly. A few elongate setae arise on the trunk.
(34) Specimen 6673 (PED 0871 spec 22) is only accessible from the lateral right side of the body (Figure 10A,B). It is preserved in the same amber piece as specimens 6652–6664 and 6668–6672. The specimen shows the overall slender morphology known for older larvae of Nevrorthidae in the anterior region, but especially the posterior trunk appears much less slender, the segments of the abdomen are extremely short. The amber is very clear and the overall body outline is well accessible. Head with stout antennae, stylets, and labial palps. Antennae subdivided into three elements with a few setae apparent. Stylets with a broad proximal region tapering distally. Labial palps are subdivided into four elements. Trunk appendages with five major elements (coxa, trochanter, femur, tibia, tarsus; Figure 10B), well preserved, but they are entangled with each other, partly obscuring subdivisions and details. Coxa appears elongate and tubular; trochanter similar, but way shorter; femur similar to coxa, but a bit shorter; tibia similar and shorter as well; tarsus similar too, but a bit longer than tibia and taper distally with a pair of claws at the end. The thorax is subdivided into pro-, meso-, and metathorax with a short cervix anteriorly. Prominent setae arise from the trunk appendage elements and all over the trunk. At the trunk end, the setae are longer. The insertion areas seem to be in the middle of each abdomen segment.

(35) Specimen 6674 (PED 0871 spec 23) is only accessible from the lateral right side of the body (Figure 10C,D). It is preserved in the same amber piece as specimens 6652–6664 and 6668–6673. The specimen shows the overall slender morphology known for older larvae of Nevrorthidae in the anterior region, but especially the posterior trunk appears much less slender, the segments of the abdomen are extremely short. The overall body outline is partly obscured by bubbles, but still accessible. Head with antennae, stylets, and labial palps. One of the antennae is broken, the other one is elongated and subdivided into three elements. Stylets tapering distally. The labial palps are not accessible in this view. Trunk appendages with five major elements (coxa, trochanter, femur, tibia, tarsus; Figure 10D). The thorax is subdivided into pro-, meso-, and metathorax with a short cervix anteriorly. Numerous elongate setae arise from the ten abdomen units. The insertion areas seem to be in the middle of each abdomen segment.
(36) Specimen 6676 (PED 0871 spec 25) is only accessible from the lateral right side of the body (Figure 10E,F). It is preserved in the same amber piece as specimens 6652–6664 and 6668–6674. The specimen shows the overall slender morphology known for older larvae of Nevrorthidae in the anterior region, but especially the posterior trunk appears much less slender, the segments of the abdomen are extremely short. Overall body outline well accessible. Head with stout antennae, stylets, and labial palps. Antennae subdivided into three elements. Stylets tapering distally. Labial palps look similar to the antennae, subdivided into at least three elements apparent, proximal region not accessible. Trunk appendages with five major elements (coxa, trochanter, femur, tibia, tarsus; Figure 10F), well preserved, but they are entangled with each other, partly obscuring subdivisions and details. Coxa and femur are both tubular and elongated; tibia similar, but shorter than femur; tarsus similar, but a bit longer than tibia, distally with a pair of claws. The thorax is clearly subdivided into pro-, meso-, and metathorax with a short cervix anteriorly. The abdomen is subdivided into 10 units with several setae. They seem to have their insertion areas in the middle of each unit and are longer at the trunk end. Also, the thorax appendages bear several setae.
(37) Specimen 6677 (PED 0871 spec 26) is only partly accessible from the lateral left side of the body (Figure 11E,F). It is preserved in the same amber piece as specimens 6652–6664, 6668–6674 and 6676. The specimen shows the overall slender morphology known for older larvae of Nevrorthidae in the anterior region, but especially the posterior trunk appears much less slender, the segments of the abdomen are extremely short. The body outline is well accessible, except for the head with the appendages. The anterior part of the head is outside the edge of the amber. Trunk appendages with five major elements (coxa, trochanter, femur, tibia, tarsus; Figure 11F), well preserved, but they are entangled with each other, partly obscuring subdivisions and details. The thorax is clearly subdivided into pro-, meso-, and metathorax with a short cervix anteriorly. Abdomen subdivided into 10 units. Trunk with several setae.
(38) Specimen 6678 (PED 0871 spec 27) is only partly accessible from the lateral left side of the body (Figure 11C,D). It is preserved in the same amber piece as specimens 6652–6664, 6668–6674, 6676 and 6777. The specimen shows the overall slender morphology known for older larvae of Nevrorthidae in the anterior region, but especially the posterior trunk appears much less slender, the segments of the abdomen are extremely short. The body outline is well accessible, except for the head with the appendages. The anterior part of the head outside the edge of the amber. Trunk appendages with five major elements (coxa, trochanter, femur, tibia, tarsus; Figure 11D). Coxa and femur are both tubular and elongated; tibia similar, but shorter than femur; tarsus similar, but a bit longer than tibia; distally one claw remains. The thorax is subdivided into pro-, meso-, and metathorax with a short cervix anteriorly. The abdomen is subdivided into 10 units. Several setae arise at the middle of each unit.

(39) Specimen 6679 (PED 0871 spec 28) is only accessible from the dorsal side of the body (Figure 11I) and sits very deep within the amber piece. As the amber piece contains multiple inclusions it is not possible to grind it further down. It is preserved in the same amber piece as specimens 6652–6664, 6668–6674, and 6676–6678. The specimen shows the overall slender morphology known for older larvae of Nevrorthidae in the anterior region, but especially the posterior trunk appears much less slender, the segments of the abdomen are extremely short. Most parts of the head, thorax, and trunk appendages are covered by other inclusions. At the abdomen, several setae arise.
(40) Specimen 6680 (PED 0871 spec 29) is only accessible from the lateral right side of the body (Figure 11A,B). It is preserved in the same amber piece as specimens 6652–6664, 6668–6674, and 6676–6679. The specimen shows the overall slender morphology known for older larvae of Nevrorthidae in the anterior region, but especially the posterior trunk appears much less slender, the segments of the abdomen are extremely short. The amber is very clear, hence the overall body outline of the specimen is well accessible. Head with antennae, stylets, and labial palps. Stout antennae are elongated and show subdivision into three elements. Stylets with a broad proximal region, curved and tapering distally. Labial palps are slightly shorter than antennae, subdivided into at least three elements apparent, proximal region not accessible. Trunk appendages with five major elements (coxa, trochanter, femur, tibia, tarsus; Figure 11B), well preserved, but they are entangled with each other, partly obscuring subdivisions and details. Coxa is tubular and elongated; trochanter conical and short; femur similar to coxa, but shorter; tibia similar as well, but shorter than femur; tarsus tubular as well, but a bit longer than tibia and tapering distally with a pair of claws. The thorax is clearly subdivided into pro-, meso-, and metathorax with a short cervix anteriorly. Abdomen subdivided into 10 units. Numerous setae arise from the body. They seem to have their insertion areas in the middle of each unit and get longer at the trunk end. Also, the thorax appendages bear several setae.
(41) Specimen 6681 (PED 0871 spec 30) is only accessible from the lateral left side of the body (Figure 11G,H). It is preserved in the same amber piece as specimens 6652–6664, 6668–6674, and 6676–6680. The specimen shows the overall slender morphology known for older larvae of Nevrorthidae in the anterior region, but especially the posterior trunk appears much less slender, the segments of the abdomen are extremely short. As the amber piece contains multiple inclusions most parts of the head and thorax are obscured. Head with one antenna, stylets, and labial palps. The stout antenna is subdivided into three elements. Stylets with a broad proximal region, slightly curved and tapering distally. The labial palps are mostly covered by the stylets. Trunk appendages with five major elements (coxa, trochanter, femur, tibia, tarsus; Figure 11H), well preserved, but they are entangled with each other, partly obscuring subdivisions and details. Several setae arise from the ten abdomen units. Most insertion areas are obscured by Verlumung.
(42) Specimen 6682 (PED 0871 spec 31) is only partly accessible from the lateral left side of the body (Figure 11J upper and lower). It is preserved in the same amber piece as specimens 6652–6664, 6668–6674, and 6676–6681. The specimen shows the overall slender morphology known for older larvae of Nevrorthidae in the anterior region, but especially the posterior trunk appears much less slender, the segments of the abdomen are extremely short. The body outline is well accessible, except for the head and the trunk appendages. The anterior part of the specimen is outside the edge of the amber. The abdomen is subdivided into 10 units and bears several setae.
(43) Specimen 6665 (PED 0871 spec 14) is accessible from the lateral left and right side (Figure 12A,B) and sits very deep within the amber piece. It is preserved in the same amber piece as specimens 6652–6664, 6668–6674, and 6676–6682. As the amber piece contains multiple inclusions it is not possible to grind it further down. Slightly more accessible parts include the anterior region of the specimen and the trunk appendages distal with the pair of claws. Other aspects are not accessible. Most of the posterior body is obscured by various inclusions.

(44) Specimen 6666 (PED 0871 spec 15) is accessible from the lateral left and right side (Figure 12E,F) and sits very deep within the amber piece. It is preserved in the same amber piece as specimens 6652–6665, 6668–6674, and 6676–6682. As the amber piece contains multiple inclusions it is not possible to grind it further down. The most accessible parts are the distal end of one trunk appendage and a few units of the abdomen. Other aspects are not accessible. The remaining parts of the body are obscured by various inclusions.
(45) Specimen 6667 (PED 0871 spec 16) is only accessible from the dorsal side (Figure 12C) and sits very deep within the amber piece. It is preserved in the same amber piece as specimens 6652–6666, 6668–6674, and 6676–6682. As the amber piece contains multiple inclusions it is not possible to grind it further down. The most accessible parts are the thorax segments and a few units of the anterior abdomen. Other aspects are not well accessible. The remaining parts of the body are obscured by various inclusions.
(46) Specimen 6675 (PED 0871 spec 24) is only accessible from the lateral left side (Figure 12D) and sits very deep within the amber piece. It is preserved in the same amber piece as specimens 6652–6674 and 6676–6682. As the amber piece contains multiple inclusions it is not possible to grind it further down. The slightly more accessible part is the head with appendages. Other aspects are not well accessible. The remaining parts of the body are mostly obscured by various inclusions. Nevertheless, the specimen shows the overall slender morphology known for older larvae of Nevrorthidae in the anterior region, especially the posterior trunk, but appears much less slender, the segments of the abdomen are extremely short.
(47) Specimen 6683 (PED 1373 spec 1) is only accessible from the lateral right side of the body (Figure 13A,B). The specimen shows the overall slender morphology known for older larvae of Nevrorthidae in the anterior region, but especially the posterior trunk appears much less slender, the segments of the abdomen are extremely short. The body outline is well accessible. Head with one elongate antenna, both stylets, and one labial palp (left). Antenna subdivided into three elements. Stylets with a broad proximal region tapering distally. Labial palp is subdivided into at least two elements apparent, proximal region not accessible. Trunk appendages with five major elements (coxa, trochanter, femur, tibia, tarsus; Figure 13B), well preserved, but they are entangled with each other, partly obscuring subdivisions and details. The thorax is subdivided into pro-, meso-, and metathorax with a short cervix anteriorly. Several setae arise from the abdomen.

(48) Specimen 6684 (PED 1373 spec 2) is only accessible from the lateral left side of the body (Figure 13E) and sits very deep within the amber piece. Hence, the specimen is not fully in focus. It is preserved in the same amber piece as specimen 6683. Subdivisions of the body are not visible. Nevertheless, the specimen shows the overall slender morphology known for older larvae of Nevrorthidae in the anterior region, but especially the posterior trunk appears much less slender, the segments of the abdomen are extremely short. Head with one elongate antenna, both stylets, and one labial palp (left). Stylets are curved and tapering distally. Labial palp is mainly obscured by the head. Trunk appendages are out of focus and entangled with each other. Abdomen with few setae.
(49) Specimen 6685 (PED 1373 spec 3) is only accessible from the lateral right side of the body (Figure 13F) and sits very deep within the amber piece. Hence, the specimen is not fully in focus. It is preserved in the same amber piece as specimens 6683 and 6684. Subdivisions of the body are not well visible. Some subdivisions of the thorax and abdomen are slightly accessible. Nevertheless, the specimen shows the overall slender morphology known for older larvae of Nevrorthidae in the anterior region, but especially the posterior trunk appears much less slender, the segments of the abdomen are extremely short. Head with appendages not accessible. Trunk appendages are out of focus and entangled with each other, partly obscuring subdivisions. The thorax is subdivided into pro-, meso-, and metathorax. The abdomen has no visible setae.
(50) Specimen 6686 (PED 1373 spec 4) is only accessible from the lateral right side of the body (Figure 13D) and sits very deep within the amber piece. It is preserved in the same amber piece as specimens 6683–6685. As the amber piece contains multiple inclusions it is not possible to grind it further down. The specimen shows the overall slender morphology known for older larvae of Nevrorthidae in the anterior region, but especially the posterior trunk appears much less slender, the segments of the abdomen are extremely short. The slightly better accessible parts are the head, parts of the trunk appendages and several setae, which are arising from the abdomen. From the head appendages, only one elongate antenna is accessible, subdivided into three elements. The rest of the body is mainly obscured by various inclusions.
(51) Specimen 6687 (PED 1373 spec 5) is only accessible from the ventral side of the body (Figure 13G,H). It is preserved in the same amber piece as specimen 6683–6686. The specimen shows the overall slender morphology known for older larvae of Nevrorthidae in the anterior region, but especially the posterior trunk appears much less slender, the segments of the abdomen are extremely short. The overall body outline is well accessible. Head with antennae and labial palps. The stylets are obscured by Verlumung. Antennae and labial palps are subdivided into at least four elements apparent, proximal region not accessible. Trunk appendages with five major elements (coxa, trochanter, femur, tibia, tarsus; Figure 13H), well preserved, but they are entangled with each other, partly obscuring subdivisions and details. Tarsus distally with a pair of claws. A few setae arise from the abdomen.
(52) Specimen 6688 (NHMD 115214 spec 1) is accessible from the dorsal and ventral side of the body (Figure 14 and Figure 15A–C). The specimen shows the overall slender morphology known for older larvae of Nevrorthidae in the anterior region, but especially the posterior trunk appears much less slender, the segments of the abdomen are extremely short. The amber is very clear, hence the overall body outline of the specimen is well accessible. Head with short and stout antennae, stylets, and labial palps. Antennae subdivided into three elements. Stylets are curved and tapering distally. Labial palps are mainly obscured by the stylets. Trunk appendages with five major elements (coxa, trochanter, femur, tibia, tarsus; Figure 15C), well preserved, but they are entangled with each other, partly obscuring subdivisions and details. Also, they are largely obscured by Verlumung. The thorax is subdivided into pro-, meso-, and metathorax. Several setae arise from the trunk. The insertion areas are obscured by Verlumung.
(53) Specimen 6689 (NHMD 115214 spec 2) is accessible from the dorsal and ventral side of the body (Figure 14 and Figure 15F–H). It is preserved in the same amber piece as specimen 6688. The specimen shows the overall slender morphology known for older larvae of Nevrorthidae in the anterior region, but especially the posterior trunk appears much less slender, the segments of the abdomen are extremely short. The overall body outline is well accessible. Head with stout antennae, stylets, and labial palps (Figure 15H). Antennae subdivided into at least two elements, proximal region not accessible. Stylets and labial palps are mainly obscured by Verlumung. Only the tip of the stylet and the distal element of the labial palps are visible. Trunk appendages with five major elements (coxa, trochanter, femur, tibia, tarsus; Figure 15H); these seem thicker in comparison to other specimens. The thorax is subdivided into pro-, meso-, and metathorax with a short cervix anteriorly (Figure 15G). Few setae arise from the trunk.
(54) Specimen 6690 (NHMD 115214 spec 3) is accessible from the lateral right and ventral side of the body (Figure 14 and Figure 15D,E). It is preserved in the same amber piece as specimens 6688 and 6689. The specimen shows the overall slender morphology known for older larvae of Nevrorthidae in the anterior region, but especially the posterior trunk appears much less slender, the segments of the abdomen are extremely short. The body outline is well accessible, except for the head with appendages and the thorax. The anterior part of body is largely obscured by Verlumung. Head with stout antennae, stylets, and labial palps (Figure 15D). Antennae subdivided into at least two elements apparent, proximal region not accessible. Stylets and labial palps are mostly obscured by Verlumung. Only one stylet and the distal element of the labial palps are visible. Stylet tapering distally. Trunk appendages with five major elements (coxa, trochanter, femur, tibia, tarsus), well preserved, but they are entangled with each other, partly obscuring subdivisions and details. A few setae arise from the abdomen. The insertion areas are obscured by Verlumung.
(55) Specimen 6691 (NHMD 115214 spec 4) is only accessible from the ventral side of the body (Figure 14 and Figure 15I,J). It is preserved in the same amber piece as specimens 6688–6690. The specimen shows the overall slender morphology known for older larvae of Nevrorthidae in the anterior region, but especially the posterior trunk appears much less slender, the segments of the abdomen are extremely short. The body outline is well accessible, except for the head with appendages. The head is largely obscured by Verlumung. Only the distal elements of the antennae and labial palps are visible. Stylets are entirely obscured. Trunk appendages with five major elements (coxa, trochanter, femur, tibia, tarsus; Figure 15J), well preserved, but they are entangled with each other, partly obscuring subdivisions and details. The thorax is subdivided into pro-, meso-, and metathorax. Abdomen units with several setae. They seem to have their insertion areas in the middle of each abdomen unit.
(56) Specimen 6692 (NHMD 115214 spec 5) is only accessible from the ventral side of the body (Figure 14 and Figure 15K,L). It is preserved in the same amber piece as specimens 6688–6691. The specimen shows the overall slender morphology known for older larvae of Nevrorthidae in the anterior region, but especially the posterior trunk appears much less slender, the segments of the abdomen are extremely short. The amber is very clear, hence the overall body outline of the specimen is accessible, except for parts of the head with appendages that are obscured by Verlumung. Only the distal elements of one antenna and both labial palps are visible. Stylets are entirely obscured. Trunk appendages with five major elements (coxa, trochanter, femur, tibia, tarsus; Figure 15L). Tarsus distally with a pair of claws. Abdomen units with several setae. They seem to have their insertion areas in the middle of each abdomen unit.
(57) Specimen 6693 (NHMD 115214 spec 6) is only accessible from the ventral side of the body (Figure 14 and Figure 15N). It is preserved in the same amber piece as specimens 6688–6692. The specimen shows the overall slender morphology known for older larvae of Nevrorthidae in the anterior region, but especially the posterior trunk appears much less slender, the segments of the abdomen are extremely short. The anterior part of the specimen is largely obscured by Verlumung, hence the overall body outline is partly difficult to access. Head with stout antennae, stylets, and labial palps (Figure 15N). Antennae subdivided into at least two elements, proximal region not accessible. Stylets and labial palps are mainly obscured by Verlumung. Only distal element of the labial palps is visible. Trunk appendages are mainly obscured. The most accessible part is the tarsus distally with a pair of claws. The abdomen shows a subdivision into several units. Abdomen with few setae.
(58) Specimen 6694 (NHMD 115214 spec 7) is only accessible from the ventral side of the body (Figure 14 and Figure 15M) and sits very deep within the amber piece. Hence, the specimen is not fully in focus. It is preserved in the same amber piece as specimens 6688–6693. The specimen shows the overall slender morphology known for older larvae of Nevrorthidae in the anterior region, but especially the posterior trunk appears much less slender, the segments of the abdomen are extremely short. The anterior part of the specimen is largely obscured by Verlumung, hence the overall body outline is partly difficult to access. Head with antennae, stylets, and labial palps (Figure 15M). Only the distal elements of the antennae, both labial palps, and the tip of the stylets are accessible. Trunk appendages with five major elements (coxa, trochanter, femur, tibia, tarsus) are mostly out of focus and entangled with each other, partly obscuring subdivisions and details. Thorax and setae are not accessible. The abdomen is subdivided into several units.
(59) Specimen 6695 (CCHH 1124-4b spec 1) is only accessible from the lateral right side of the body (Figure 16 and Figure 17A,B). The specimen shows the overall slender morphology known for older larvae of Nevrorthidae in the anterior region, but especially the posterior trunk appears much less slender, the segments of the abdomen are extremely short. The specimen is a bit curved dorsally, basically adopting a U-shaped posture. Overall body outline well accessible. Head with antennae and stylets. Antennae are elongated and subdivided into three elements. Stylets are curved and tapering distally. Labial palps are not accessible. Trunk appendages with five major elements (coxa, trochanter, femur, tibia, tarsus; Figure 17B). Coxa is tubular and elongated; trochanter not accessible; femur similar to coxa, but shorter; tibia similar, but shorter than femur; tarsus similar and taper distally with a pair of claws at the end. The thorax is subdivided into pro-, meso-, and metathorax. Numerous elongate setae arise from the abdomen. The insertion areas seem to be in the middle of each abdomen unit.

(60) Specimen 6696 (CCHH 1124-4b spec 2) is only accessible from the lateral left side of the body (Figure 16 and Figure 17C,D). It is preserved in the same amber piece as specimen 6695. The specimen shows the overall slender morphology known for older larvae of Nevrorthidae in the anterior region, but especially the posterior trunk appears much less slender, the segments of the abdomen are extremely short. The specimen is extremely curved ventrally, basically adopting a U-shaped posture. Overall body outline well accessible. Head with antennae, stylets, and labial palps. Antennae are elongated and subdivided into three elements. Stylets are not well accessible, because of the position of the head, but tapering distally. The labial palps are mostly covered by the stylets. Trunk appendages with five major elements (coxa, trochanter, femur, tibia, tarsus; Figure 17D) are entangled with each other, partly obscuring subdivisions and details. The thorax is subdivided into pro-, meso-, and metathorax, with a short cervix anteriorly. Numerous elongate setae arise from the trunk. They seem to be longer at the trunk end.
(61) Specimen 6697 (CCHH 1124-4b spec 3) is only accessible from the dorsal side of the body (Figure 16 and Figure 17E,F). It is preserved in the same amber piece as specimens 6695 and 6696. The specimen shows the overall slender morphology known for older larvae of Nevrorthidae in the anterior region, but especially the posterior trunk appears much less slender, the segments of the abdomen are extremely short. Despite several inclusions, the overall body outline is well accessible, except for the head appendages. Head with elongate antennae and stylets. Subdivision of the antennae are not accessible. Stylets proximally straight, but sharply curved more distally and tapering at the end. Labial palps and trunk appendages are not accessible. The thorax is subdivided into pro-, meso-, and metathorax with a short cervix anteriorly. Few elongate setae arise from the abdomen.
(62) Specimen 6698 (CCHH 1124-4b spec 4) is only accessible from the dorsal side of the body (Figure 16 and Figure 17G) and sits very deep within the amber piece. Hence, the specimen is not fully in focus. As the amber piece contains multiple inclusions it is not possible to grind it further down. It is preserved in the same amber piece as specimens 6695–6697. The specimen shows the overall slender morphology known for older larvae of Nevrorthidae in the anterior region, but especially the posterior trunk appears much less slender, the segments of the abdomen are extremely short. Overall body outline accessible. Head with elongate antennae and stylets. Stylets proximally straight, but sharply curved more distally and tapering at the end. Labial palps and trunk appendages are not well accessible. The thorax is subdivided into pro-, meso-, and metathorax with a short cervix anteriorly. Several setae arise from the trunk.
(63) Specimen 6699 (CCHH 1124-4b spec 5) is only accessible from the lateral right side of the body (Figure 16 and Figure 17H) and sits very deep within the amber piece. Hence, the specimen is not fully in focus. As the amber piece contains multiple inclusions it is not possible to grind it further down. It is preserved in the same amber piece as specimens 6695–6698. The specimen shows the overall slender morphology known for older larvae of Nevrorthidae in the anterior region, but especially the posterior trunk appears much less slender, the segments of the abdomen are extremely short. Overall outline is difficult to access. The slightly better accessible parts of the specimen are the abdomen with several setae and the trunk appendages distally with a pair of claws.
(64) Specimen 6700 (CCHH 1124-4b spec 6) is only accessible from the lateral right side of the body (Figure 16 and Figure 17I) and sits very deep within the amber piece. Hence, the specimen is out of focus. As the amber piece contains multiple inclusions it is not possible to grind it further down. It is preserved in the same amber piece as specimens 6695–6699. The specimen shows the overall slender morphology known for older larvae of Nevrorthidae in the anterior region, but especially the posterior trunk appears much less slender, the segments of the abdomen are extremely short. Overall outline is difficult to access. The slightly better accessible parts of the specimen are the trunk appendages and the abdomen with several setae.
(65) Specimen 6701 (CCHH 1270-4 spec 1) is only accessible from the lateral left side of the body (Figure 18 and Figure 19A–C). The specimen shows the overall slender morphology known for older larvae of Nevrorthidae (Figure 19A,B). Overall outline is well accessible, yet a few details of the anterior part of the abdomen are obscured by a large bubble, and from the sixth segment of the abdomen the posterior part of the specimen is missing. The entire specimen, except for the head and parts of the trunk appendages, appears covered with pyrite. Head with antennae, stylets, and labial palps. Antenna is elongated and shows subdivision into numerous elements (about 18). Stylets taper distally; they are straight and broad proximally, but sharply curved more distally. Labial palps are shorter than antennae, subdivided into three elements (Figure 19C). Trunk appendages with five major elements (coxa, trochanter, femur, tibia, tarsus; Figure 19B), well preserved, but they are entangled with each other, partly obscuring subdivisions and details. Well preserved are the pairs of claws distally at the tarsi. The thorax is subdivided into pro-, meso-, and metathorax with a longer cervix anteriorly. Prominent setae arise from the appendage elements, exact arrangement is difficult to assess. Trunk segments with prominent long setae; insertion areas obscured.

(66) Specimen 6702 (CCHH 1270-4 spec 2) is well accessible from the lateral left and right side (Figure 18 and Figure 20A–C) and less well from the dorsal side of the body (Figure 20D,E). It is preserved in the same amber piece as specimen 6701. The specimen shows the overall slender morphology known for older larvae of Nevrorthidae. Overall body outline well accessible. Head with antennae, stylets, and labial palps. Antenna is elongated and shows subdivision into numerous elements (about 13). Stylets are curved and taper distally. Labial palps are slightly shorter than antennae, subdivided into four elements (Figure 20B). Trunk appendages with five major elements (coxa, trochanter, femur, tibia, tarsus; Figure 20B). Coxa is tubular and elongated; trochanter not accessible; femur similar to coxa, but shorter; tibia similar to femur, but thinner; tarsus quite slender again and tapering distally with a pair of claws. The thorax is subdivided into pro-, meso-, and metathorax with a longer cervix anteriorly. Abdomen with at least eight units. The cervix and prothorax show a longitudinal groove (Figure 20E). The two thorax segments posterior to the prothorax with a distinct pattern of dorsal sclerotisations. After the anterior third of the segment, a distinct fold is apparent, subdividing the dorsal region of each segment into two distinct subregions (Figure 20B). Prominent setae arise from the trunk appendage elements. Femur and tibia are distally with a set of three setae each. Few, less prominent setae also apparent further proximally, no clear pattern apparent. Also, a few setae arise from the trunk; insertion areas are obscured.

(67) Specimen 6703 (CCHH 1387-1) is well accessible from the ventral, dorsal, and lateral right side (Figure 21A–D). The specimen shows the overall slender morphology known for older larvae of Nevrorthidae. Overall body outline well accessible. Head with antennae, stylets, and labial palps. Antenna is elongated and shows subdivision into numerous elements (about 15; Figure 21E). Stylets taper distally; they are straight proximally, but sharply curved more distally. Labial palps are shorter than antennae, subdivided into four elements (Figure 21A). Trunk appendages with five major elements (coxa, trochanter, femur, tibia, tarsus; Figure 21A,B). Coxa is tubular and thickened; trochanter tubular, elongated, and short; femur similar to trochanter, but longer and thicker; tibia similar to femur, but shorter and thinner; tarsus quite slender and tapering distally with a pair of claws. The thorax is subdivided into pro-, meso-, and metathorax with a longer cervix anteriorly. The cervix and prothorax have a longitudinal groove (Figure 21E). The trunk segments posterior to the prothorax show a distinct pattern of dorsal sclerotisation. After the anterior third of the segment, a distinct fold is apparent, subdividing the dorsal region of each segment into two distinct subregions (Figure 21B,C). Prominent setae arise from the trunk appendage elements. Femur and tibia are distally with a set of three setae each. Trunk segments with prominent long setae. These arise close to the posterior end of each segment and appear to form a kind of ring around the segment. At the abdomen units appear to be six setae dorsally and four ventrally.

(68) Specimen 6704 (SMF Be 2192) is only accessible from the lateral left side of the body (Figure 22G) and sits very deep within the amber piece. Hence, the specimen is not fully in focus. As the amber piece contains multiple inclusions it is not possible to grind it further down. The specimen shows the overall slender morphology known for older larvae of Nevrorthidae in the anterior region, but especially the posterior trunk appears much less slender, the segments of the abdomen are extremely short. The overall body outline is accessible. Head with antennae, stylets, and labial palps. Further details of the head appendages are mainly obscured. Stylets taper distally. Trunk appendages with five major elements (coxa, trochanter, femur, tibia, tarsus; Figure 22G) are entangled with each other, partly obscuring subdivisions and details. The thorax is subdivided into pro-, meso-, and metathorax with a short cervix anteriorly. Several setae arise from the trunk.

(69) Specimen 6705 (PED 0792) is accessible from the lateral right and left side (Figure 22A–D) and sits very deep within the amber piece. As the amber piece contains multiple inclusions it is not possible to grind it further down. Additionally, the head is accessible from the dorsal side (Figure 22B). The specimen shows the overall slender morphology known for older larvae of Nevrorthidae. The overall body outline is accessible, yet most details, especially of the trunk are obscured by Verlumung and numerous bubbles. Head with antennae, stylets, and labial palps. Antenna is elongated and shows subdivision into numerous elements (about 11; Figure 22C). Stylets taper distally; they are straight proximally, but sharply curved more distally. Labial palps are slightly shorter than antennae, subdivided into at least four elements (Figure 21A), proximal region is not accessible. Trunk appendages with five major elements (coxa, trochanter, femur, tibia, tarsus; Figure 22D). Proximal region with coxa and trochanter mainly obscured by Verlumung. Femur tubular and elongated; tibia similar to femur, but shorter; tarsus quite slender and, but longer than tibia, tapering distally with a pair of claws. Prominent setae arise from the trunk appendage elements. Femur and tibia are distally with a set of three setae each. Also, several setae arise from the trunk; insertions are obscured.
(70) Specimen 6706 (PED 1379) is well accessible from the ventral and dorsal side (Figure 23A–C). The specimen shows the overall slender morphology known for older larvae of Nevrorthidae in the anterior region, but especially the posterior trunk appears much less slender, the segments of the abdomen are extremely short. The amber is very clear and the overall outline is well accessible. Head with short and stout antennae, stylets, and labial palps. Antennae are subdivided into three elements. Stylets are curved and tapering distally. Labial palps are subdivided into four elements (Figure 23B). Trunk appendages with five major elements (coxa, trochanter, femur, tibia, tarsus), well preserved, but they are entangled with each other, partly obscuring subdivisions and details. Coxa tubular thickened and elongated; trochanter not accessible; femur similar to coxa, but shorter; tibia similar to femur, but shorter again; tarsus similar to tibia, but a bit longer and tapering distally with a pair of claws. The thorax is subdivided into pro-, meso-, and metathorax with a short cervix anteriorly. Abdomen with 10 units. Prominent setae arise from the trunk appendage elements and several elongate setae arise from the trunk.

### 3.4. Additional Fossil Larval Representatives of Nevrorthidae in Kachin amber

**Figure 24:**
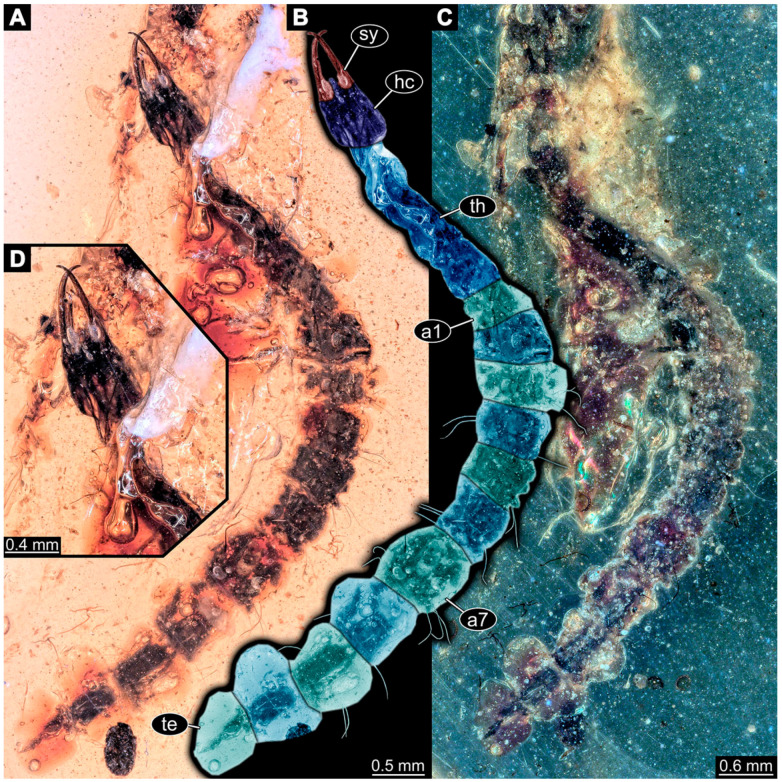
Nevrorthidae; fossil larva preserved in Kachin amber, specimen 6707 (BUB 3703), ring light. (**A**). Dorsal view. (**B**). Colour-marked version of (**A**). (**C**). Image flipped, ventral view. (**D**). Close-up of head. Abbreviations: a1–7 = abdomen segments 1–7; hc = head capsule; sy = stylet; te = trunk end; th = thorax.

**Figure 25:**
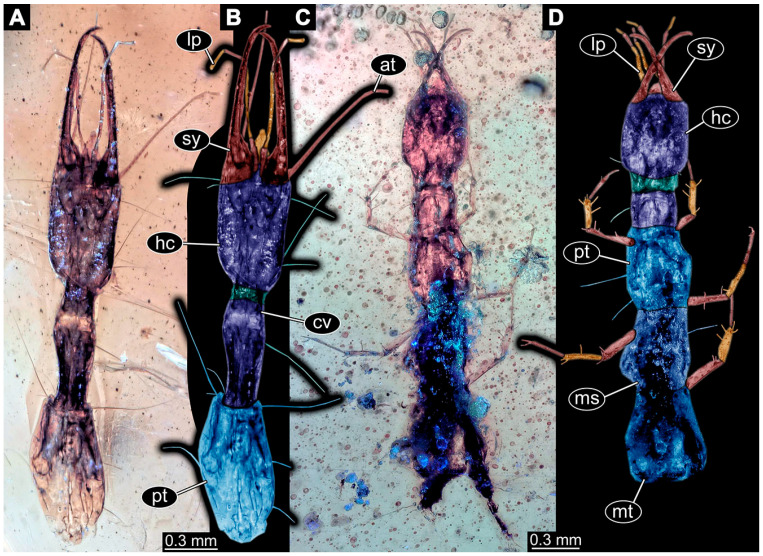
Nevrorthidae; fossil larvae preserved in Kachin amber. (**A**,**B**). Specimen 6708 (PED 0259). (**A**). Dorsal view, ring light. (**B**). Colour-marked version of (**A**). (**C**,**D**). Specimen 6709 (PED 0327). (**C**). Dorsal view, cross-polarised light. (**D**). Colour-marked version of (**C**). Abbreviations: at = antenna; cv = cervix (neck); hc = head capsule; lp = labial palp; ms = mesothorax; mt = metathorax; pt = prothorax; sy = stylet.

**Figure 26:**
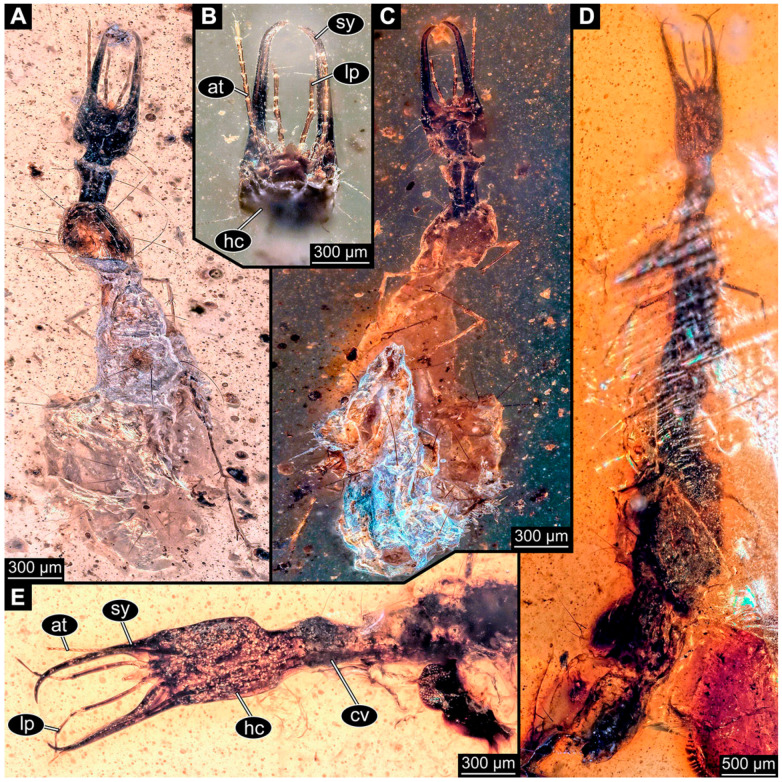
Nevrorthidae; fossil larvae preserved in Kachin amber, ring light. (**A**–**C**). Specimen 6710 (PED 0632). (**A**). Dorsal view. (**B**). Close-up of head. (**C**). Ventral view. (**D**,**E**). Specimen 6711 (PED 0663). (**D**). Ventral view, image flipped. (**E**). Close-up of head. Abbreviations: at = antenna; cv = cervix (neck); hc = head capsule; lp = labial palp; sy = stylet.

**Figure 27:**
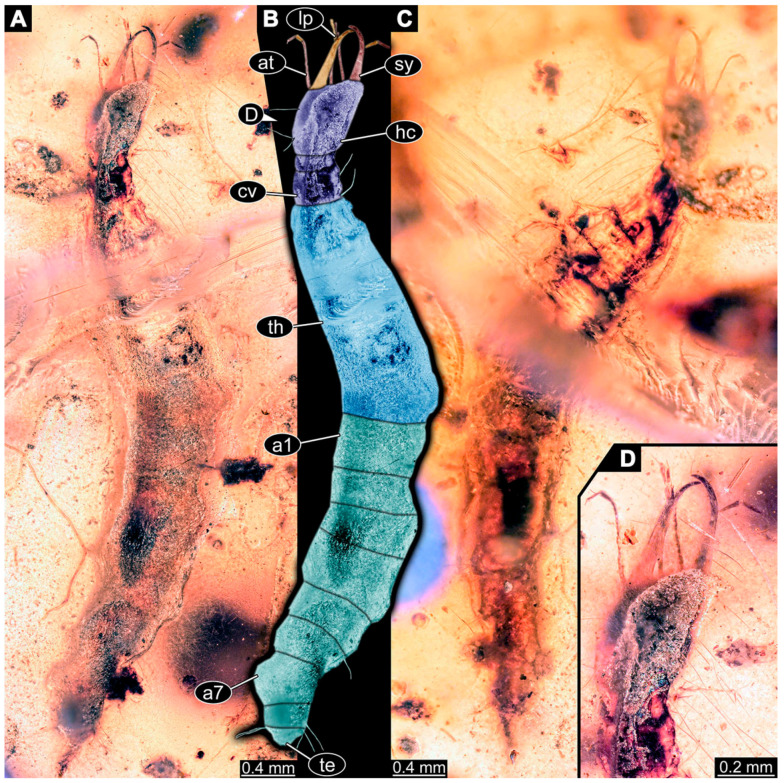
Nevrorthidae; fossil larva preserved in Kachin amber; specimen 6712 (PED 1338), ring light. (**A**). Dorsal view. (**B**). Colour-marked version of (**A**). (**C**). Ventral view. (**D**). Close-up of head. Abbreviations: a1–7 = abdomen segments 1–7; at = antenna; cv = cervix (neck); hc = head capsule; lp = labial palp; sy = stylet; te = trunk end; th = thorax.

**Figure 28:**
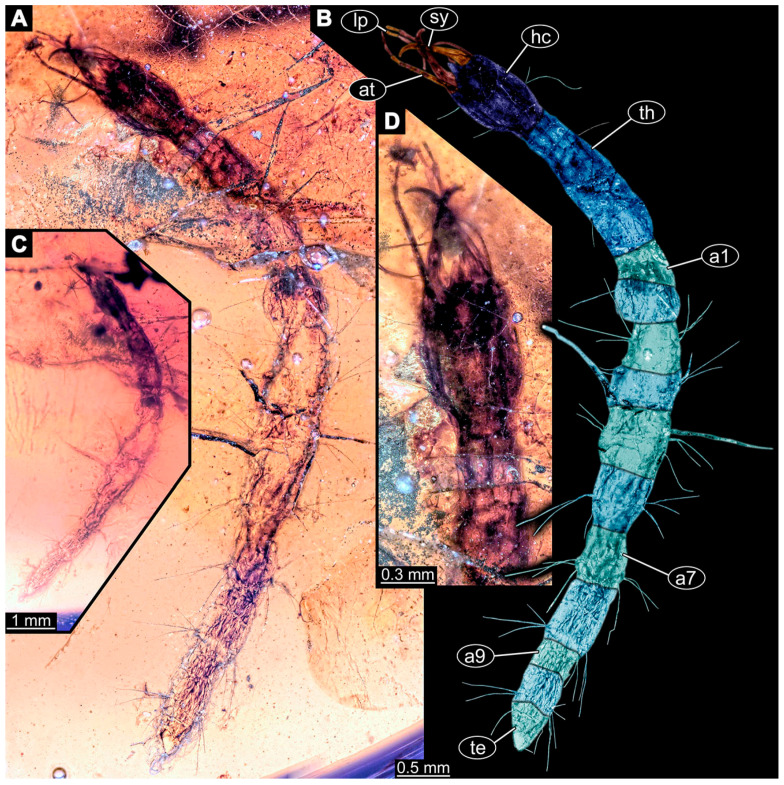
Nevrorthidae; fossil larva preserved in Kachin amber; specimen 6714 (PED 2447), ring light. (**A**). Dorsal view. (**B**). Colour-marked version of (**A**). (**C**). Ventral view, image flipped. (**D**). Close-up of head. Abbreviations: a1–9 = abdomen segments 1–9; at = antenna; hc = head capsule; lp = labial palp; sy = stylet; te = trunk end; th = thorax.

**Figure 29:**
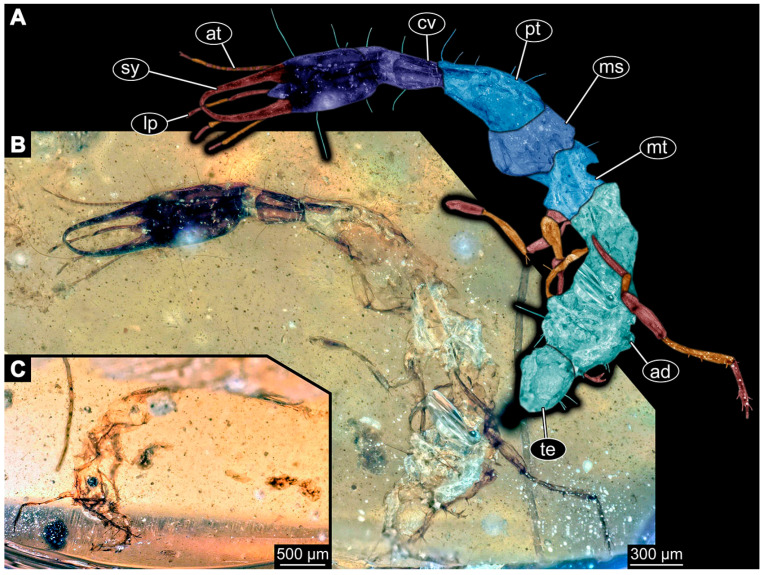
Nevrorthidae; fossil larva preserved in Kachin amber; specimen 6715 (PED 2622), ring light. (**A**). Colour-marked version of (**B**). (**B**). Dorsal view. (**C**). Ventral view. Abbreviations: ad = abdomen; at = antenna; cv = cervix (neck); lp = labial palp; ms = mesothorax; mt = metathorax; pt = prothorax; sy = stylet; te = trunk end.

**Figure 30:**
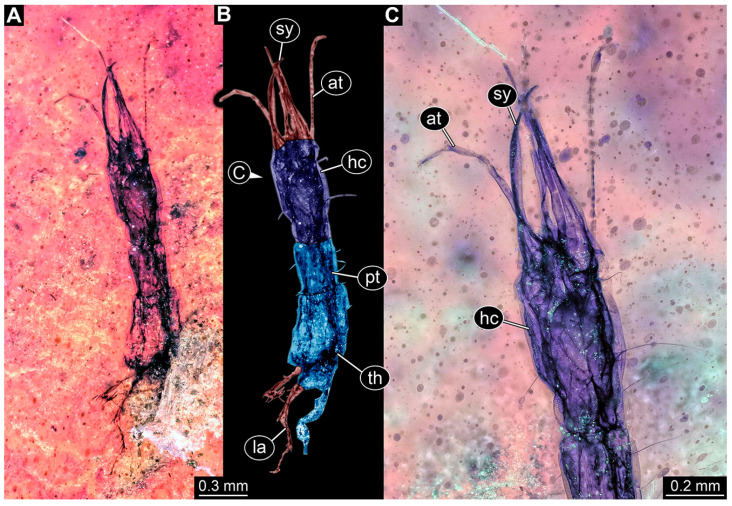
Nevrorthidae; fossil larva preserved in Kachin amber; specimen 6718 (PED 2744), ring light. (**A**). Dorsal view. (**B**). Colour-marked version of (**A**). (**C**). Close-up of head. Abbreviations: at = antenna; hc = head capsule; la = locomotory appendage (leg); pt = prothorax; sy = stylet; th = thorax.

**Figure 31:**
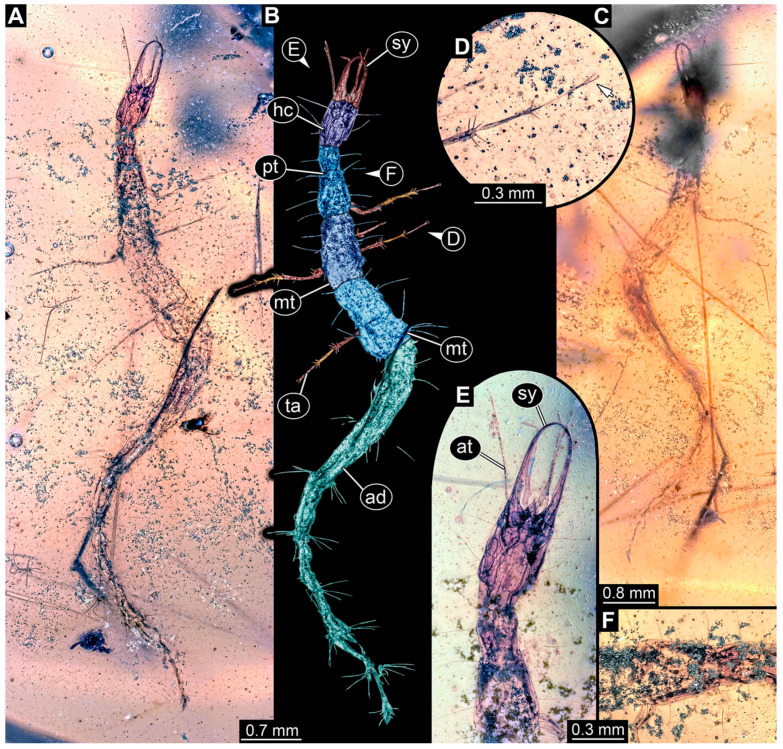
Nevrorthidae; fossil larva preserved in Kachin amber; specimen 6719 (PED 2786). (**A**). Dorsal view. (**B**). Colour-marked version of (**A**). (**C**). Ventral view. (**D**). Close-up of tarsus; arrow marks tarsal claws. (**E**). Close-up of head. (**F**). Close-up of prothorax. (**A**,**C**,**F**). Ring light. (**E**). Cross-polarised light. Abbreviations: ad = abdomen; at = antenna; hc = head capsule; ms = mesothorax; mt = metathorax; pt = prothorax; sy = stylet; ta = tarsus.

**Figure 32:**
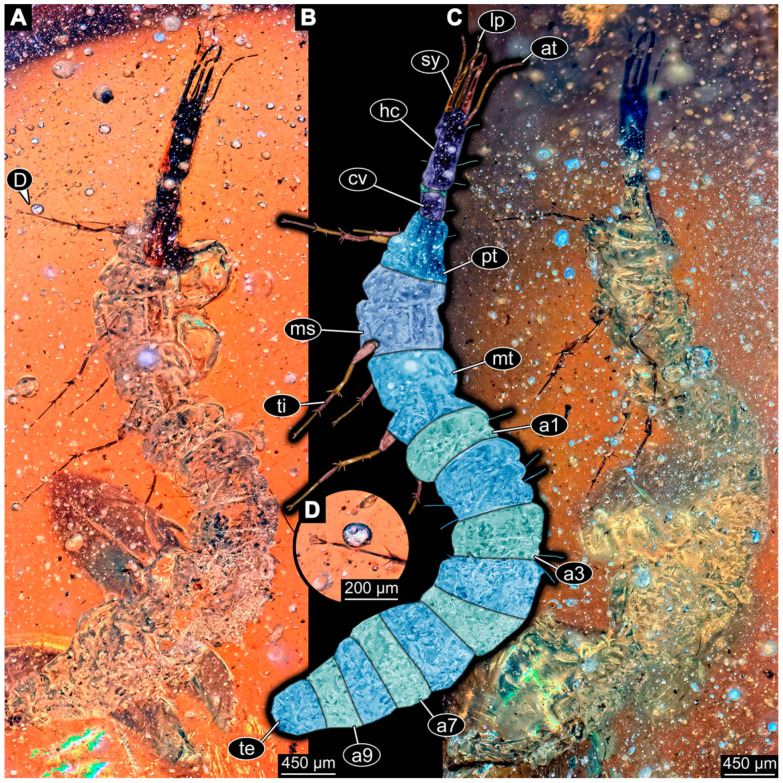
Nevrorthidae; fossil larva preserved in Kachin amber; specimen 6720 (Weiterschan BuB 24), ring light; all images flipped. (**A**). Lateral view, right. (**B**). Colour-marked version of (**A**). (**C**). Lateral view, right. (**D**). Close-up of tarsus region. Abbreviations: a1–9 = abdomen segments 1–9; at = antenna; cv = cervix (neck); hc = head capsule; lp = labial palp; ms = mesothorax; mt = metathorax; pt = prothorax; sy = stylet; ta = tarsus; te = trunk end; ti = tibia.

(71) Specimen 6707 (BUB 3703) is only accessible from the dorsal side of the body (Figure 24A–C). The specimen shows the overall slender morphology known for older larvae of Nevrorthidae. The overall body outline is partly accessible, except for the labial palps, the trunk appendages, and the thorax subdivisions. From the head, only antennae and stylets are accessible. Antennae are short and elongated. Stylets are curved and tapering distally. Several elongate setae arise from the abdomen; insertion areas are obscured.

(72) Specimen 6708 (PED 0259) is only accessible from the dorsal side of the body (Figure 25A,B). The specimen shows the overall slender morphology known for older larvae of Nevrorthidae. Of this specimen, only the head with appendages, the cervix, and prothorax are preserved. Head with antennae, stylets, and labial palps. Antenna is elongated and shows subdivision into numerous elements (about 21; Figure 25A). Stylets taper distally; they are straight proximally, but sharply curved more distally. Labial palps are slightly shorter than antennae, subdivided into four elements (Figure 25B). The long cervix and prothorax show a longitudinal groove (Figure 25A). A few elongated setae arise from the trunk.

(73) Specimen 6709 (PED 0327) is only accessible from the dorsal side of the body (Figure 25C,D). The specimen shows the overall slender morphology known for older larvae of Nevrorthidae. Of this specimen, the entire abdomen is missing. Head with antennae, stylets, and labial palps. Antenna is elongated and subdivided into at least two elements, proximal region is not accessible. The same applies to the labial palps, but there are three elements apparent. Stylets taper distally; they are straight proximally, but curved more distally. Trunk appendages with five major elements (coxa, trochanter, femur, tibia, tarsus). Coxa and trochanter are not accessible from the dorsal side of the body. Femur tubular and elongated; tibia similar to femur, but shorter; tarsus quite slender and tapering distally with a pair of claws. The thorax is subdivided into pro-, meso-, and metathorax with a short cervix anteriorly. A few elongated setae arise from the trunk.
(74) Specimen 6710 (PED 0632) is accessible from the ventral and dorsal side (Figure 26A–C). The specimen shows the overall slender morphology known for older larvae of Nevrorthidae. Of this specimen, the entire abdomen is missing and the posterior part of the thorax is mainly destroyed and obscured by Verlumung. Head with antennae, stylets, and labial palps. Antenna is elongated and shows subdivision into numerous elements (about 12; Figure 26B). Stylets taper distally; they are straight proximally, but sharply curved more distally. Labial palps are slightly shorter than antennae, subdivided into four elements (Figure 26B). Trunk appendages with five major elements (coxa, trochanter, femur, tibia, tarsus), but mostly destroyed or obscured. Most accessible are the tarsi distal with a pair of claws. Subdivision of the thorax is obscured by Verlumung. This specimen has a long cervix and several elongate setae arise from the trunk.

(75) Specimen 6711 (PED 0663) is only accessible from the ventral side of the body (Figure 26D,E) and sits very deep within the amber piece. The specimen shows the overall slender morphology known for older larvae of Nevrorthidae. The most accessible parts of the specimen are the head with appendages and the long cervix. Head with antennae, stylets, and labial palps. Antenna is elongated and shows subdivision into numerous elements (Figure 26E). Stylets taper distally; they are straight proximally, but sharply curved more distally. Labial palps are slightly longer than antennae, subdivided into four elements (Figure 26E). Trunk appendages are mostly obscured, as well as the subdivisions of the thorax and anterior part of the abdomen. Few setae arise from the trunk insertion areas obscured.
(76) Specimen 6712 (PED 1338) is only accessible from the dorsal side of the body (Figure 27A–C) and sits very deep within the amber piece. As the amber piece contains multiple inclusions it is not possible to grind it further down. The specimen shows the overall slender morphology known for older larvae of Nevrorthidae. The overall body outline is accessible. Most accessible parts of the specimen are the head with appendages and the short cervix. Head with antennae, stylets, and labial palps. Antenna is elongated and shows subdivision into numerous elements (Figure 27D). Stylets are curved and taper distally. Only the distal two elements of the labial palps are apparent, the proximal region is obscured by the stylets. Despite the ventral view of the specimen the trunk appendages are not well accessible. Subdivision of the thorax is obscured by other inclusions. Trunk with few elongate setae.

(77) Specimen 6713 (PED 2001) is only accessible from the dorsal side of the body (Figure 22E,F) and sits very deep within the amber piece. As the amber piece contains multiple inclusions it is not possible to grind it further down. The better accessible part of the specimen is the head with appendages. The rest of the body is obscured by a large black inclusion. Head with antennae, stylets, and labial palps. Antennae and labial palps are mainly obscured by inclusions. The stylets taper distally; they are straight proximally, but curved more distally. Only few fragments of the trunk appendages with a few setae are visible.
(78) Specimen 6714 (PED 2447) is accessible from the dorsal and ventral sides of the body (Figure 28A–D). The specimen shows the overall slender morphology known for older larvae of Nevrorthidae. Overall body outline well accessible, except for the trunk appendages and details of the thorax, which are obscured by various inclusions. Head with antennae, stylets, and labial palps. Antenna is elongated and shows subdivision into numerous elements (about 11, Figure 28B). Stylets taper distally; they are straight proximally, but curved more distally. Only the distal two elements of the labial palps are apparent, the proximal region is obscured by the stylets. Trunk segments with prominent long setae. These arise close to the posterior end of each segment and appear to form a kind of ring around the segment.

(79) Specimen 6715 (PED 2622) is accessible from the dorsal side of the body (Figure 29A,B) and less well from the ventral side (Figure 29C). The specimen shows the overall slender morphology known for older larvae of Nevrorthidae in the anterior region, but especially the posterior trunk appears much less slender, the abdomen is extremely short. Of this specimen, the entire abdomen and the trunk appendages are deformed. Head with antennae, stylets, and labial palps. Antenna is elongated and shows subdivision into numerous elements (about 19, Figure 29A). Stylets taper distally; they are straight proximally, but sharply curved more distally. Labial palps are subdivided into at least three elements apparent, proximal region not accessible. The thorax is subdivided into pro-, meso-, and metathorax with a longer cervix anteriorly. Few elongated setae arise from the trunk.

(80) Specimen 6716 (PED 2662) is accessible from the ventral and dorsal sides (Figure 23D,E). The specimen shows the overall slender morphology known for older larvae of Nevrorthidae. The overall outline is only accessible until the anterior part of the abdomen. The rest of the body is missing. Head with antennae, stylets, and labial palps. Subdivisions of the antennae are obscured. Stylets taper distally; they are straight proximally, but curved more distally. Labial palps are subdivided into four elements (Figure 23D). Trunk appendages are not well accessible and entangled with each other. Several setae arise from the trunk.
(81) Specimen 6717 (PED 2669) is accessible from the lateral right side of the body (Figure 12H) and the dorsal side of the head (Figure 12G). The slightly more accessible part is the head and parts of the trunk with their appendages. Other aspects are not well accessible. The remaining parts of the body are mostly obscured by various inclusions. Nevertheless, the specimen shows the overall slender morphology known for older larvae of Nevrorthidae. At the head are only the stylets and labial palps left. The stylets are curved and tapering distally. Labial palps seem to be longer than the stylets. Subdivisions are mainly obscured. A few setae arise from the body.
(82) Specimen 6718 (PED 2744) is only accessible from the dorsal side of the body (Figure 30A–C). The posterior body is missing. The specimen shows the overall slender morphology known for older larvae of Nevrorthidae. Outline of the head with appendages and anterior part of the thorax is well accessible. Head with antennae, stylets, and labial palps. Antenna is elongated and shows subdivision into numerous elements (about 19, Figure 30B). Stylets are curved and taper distally. Labial palps are subdivided into at least four elements apparent, proximal region not accessible. Few setae arise from the body.

(83) Specimen 6719 (PED 2786) is well accessible from the dorsal side of the body (Figure 31A,B) and less well from the ventral side (Figure 31C). The specimen shows the overall slender morphology known for older larvae of Nevrorthidae. The abdomen of this specimen is quite slender and deformed at the end. Overall body outline is well accessible. Head with antennae, stylets, and labial palps. Antenna is elongated and shows subdivision into numerous elements (about 13, Figure 31B). Stylets are curved and taper distally. Labial palps are subdivided into at least four elements apparent. Trunk appendages with five major elements (coxa, trochanter, femur, tibia, tarsus; Figure 31B,D). Coxa and trochanter are not accessible in this view. Femur quite slender, elongated, and tubular; tibia similar, but shorter; tarsus similar to tibia, but shorter and thinner again, tapering distally with a pair of claws. The thorax is subdivided into pro-, meso-, and metathorax with a longer cervix anteriorly. Prominent setae arise from the trunk appendage elements. Femur and tibia are distally with a set of four or five setae each. Few, less prominent setae are also apparent further proximally, no clear pattern apparent. Trunk segments with prominent long setae. These arise close to the posterior end of each segment and appear to form a kind of ring around the segment.
(84) Specimen 6720 (Weiterschan BuB 24) is only accessible from lateral right side of the body (Figure 32A–C). The specimen shows the overall slender morphology known for older larvae of Nevrorthidae. Overall body outline is well accessible. Head with antennae, stylets, and labial palps. Antenna is elongated and shows subdivision into numerous elements (about 14, Figure 31B). Stylets taper distally; they are straight proximally, but slightly curved more distally. Labial palps are subdivided into at least three elements apparent, proximal region is not accessible. Trunk appendages with five major elements (coxa, trochanter, femur, tibia, tarsus; Figure 32B,D). Coxa is tubular and elongated; trochanter not accessible in this view; femur quite slender and longer than coxa; tibia similar to femur, but shorter; tarsus similar to tibia, but longer and thinner again, tapering distally with a pair of claws. The thorax is subdivided into pro-, meso-, and metathorax with a short cervix anteriorly. Prominent setae arise from the trunk appendage elements. Femur and tibia are distally with a set of three or four setae each. Trunk segments with a few setae.

### 3.5. Shape Analysis

*Head and stylet:* The analysis resulted in four effective PCs. Principal component 1 (PC1) explains 68.0% of the overall shape variation (File S1). It is dominated by aspects of the head capsule shape on the posterior end, the size of the labrum, and the thickness at the distal tip of the stylet, as well as the position of the stylet. A low value represents a rather square-shaped head with a large labrum. The stylet is positioned on the outer part of the anterior head and gets broader at the tip. A high value represents a rather oval-shaped head without a protruding labrum. The stylet is positioned slightly more in the middle of the anterior head with a wide proximal part and is strongly tapering distally (File S2).

Principal component 2 (PC2) explains 19.1% of the overall shape variation (File S1). It is dominated by aspects of the head capsule shape on the posterior end and the broadness of the proximal part of the stylet. A low value represents a rather oval-shaped head with a large labrum and a small proximal part of the distal slightly broader stylet. A high value represents a rather square-shaped head with an extremely short labrum and a broad proximal part of the distal pointed stylet (File S2).

Principal component 3 (PC3) explains 4.1% of the overall shape variation (File S1). It is dominated by aspects of the curvature, the shape of the stylet. A low value represents a consistently strong curved stylet with a broader distal end. A high value represents a straighter distal pointed stylet with a slight curvature on the end (File S2).

Principal component 4 (PC4) explains 3.2% of the overall shape variation (File S1). It is dominated by aspects of the curvature and shape of the stylet and the labrum. A low value represents a consistently strong curved and distal broader stylet with a small proximal part and no prominent labrum visible. A high value represents a straighter and distal pointed stylet with a broad proximal part and a larger labrum (File S2).

*Stylet:* The analysis resulted in four effective PCs. Principal component 1 (PC1) explains 73.6% of the overall shape variation (File S3). It is dominated by the aspects of the curvature and the shape of the complete stylet. A low value represents a straight shape with a slightly convex outer side, and a slight curvature at the distal end. A high value represents a strong curvature over the entire stylet (File S4).

Principal component 2 (PC2) explains 10.4% of the overall shape variation (File S3). It is dominated by the aspects of the curvature and the shape of the distal tip. A low value represents a slight curvature at the distal end of the stylet. A high value represents a strong curvature at the distal end of the stylet (File S4).

Principal component 3 (PC3) explains 7.1% of the overall shape variation (File S3). It is dominated by aspects of the area in the middle and on the proximal end. A low value represents a slightly concave outer middle area and a convex proximal end, which is broader toward the inner side. A high value represents a slightly concave outer middle area and a round proximal end, which is broader toward the outer side (File S4).

Principal component 4 (PC4) explains 3.5% of the overall shape variation (File S3). It is dominated by aspects of the area on the middle and distal part of the stylet. A low value represents a consistently slight concave curved stylet, while a high value represents a straighter stylet with a slightly convex outer and concave inner side, as well as a strong curvature at the distal end (File S4).

*Head: * The analysis resulted in seven effective PCs. Principal component 1 (PC1) explains 39.7% of the overall shape variation (File S5). It is dominated by aspects of the area on the anterior and posterior ends. A low value represents a rather rectangular-shaped head without a prominent labrum. A high value represents a posterior narrowly tapered head shape and an anterior large, pointed labrum with a small proximal part (File S6).

Principal component 2 (PC2) explains 21.9% of the overall shape variation (File S5). It is dominated by aspects of the anterior area, especially the size and broadness of the labrum. A low value represents a rather rectangular-shaped head with a short, pointed labrum with a small proximal part. A high value represents a rather heart-shaped head with a large, pointed labrum with a broad proximal part (File S6).

Principal component 3 (PC3) explains 11.0% of the overall shape variation (File S5). It is dominated by aspects of the anterior area. A low value represents a rather rectangular-shaped head with a prominent labrum. A high value represents an egg-shaped head, with the labrum representing the tip of the egg shape (File S6).

Principal component 4 (PC4) explains 10% of the overall shape variation (File S5). It is dominated by aspects of the anterior and posterior areas. A low value represents a rather rectangular-shaped head capsule with a slightly concave posterior end and a pointed labrum. A high value represents a rather hexagonal-shaped head capsule, with a slightly tapered anterior and posterior end (File S6).

Principal component 5 (PC5) explains 6.0% of the overall shape variation (File S5). It is dominated by aspects of the posterior area. A low value represents a slightly concave posterior end, while a high value represents a convex posterior end (File S6).

Principal component 6 (PC6) explains 2.7% of the overall shape variation (File S5). It is dominated by aspects of the posterior area. A low value represents a rather hexagonal-shaped head capsule with a slightly tapered anterior and posterior end, while a high value represents a broader more convex head capsule shape with a pointed labrum (File S6).

Principal component 7 (PC7) explains 2.0% of the overall shape variation (File S5). It is dominated by aspects of the anterior area. A low value represents a rather broad triangular-shaped anterior end. A high value represents a rather concave antero-lateral end with a small proximal part of the labrum (File S6).

*Body with stylet:* The analysis resulted in seven effective PCs. Principal component 1 (PC1) explains 57.2% of the overall shape variation (File S7). It is dominated by the position of the stylet and the shape of the posterior trunk end. A low value represents the stylet positioned on the outer part of the head with a broad distal end, and a rather broad trunk end. A high value represents the stylet positioned slightly more in the middle of the head, and a pointed trunk end (File S8).

Principal component 2 (PC2) explains 17.0% of the overall shape variation (File S7). It is dominated by aspects of the curvature, the shape of the stylet, and the (pro-)thorax. A low value represents a rather straight distal broader stylet and a narrow (pro-)thorax. A high value represents a more curved distal pointed stylet and a rather broad (pro-)thorax (File S8).

Principal component 3 (PC3) explains 6.5% of the overall shape variation (File S7). It is dominated by aspects of the thorax shape. A low value represents a rather slender thorax, while a high value represents a rather broad thorax (File S8).

Principal component 4 (PC4) explains 5.8% of the overall shape variation (File S7). It is dominated by aspects of the curvature of the stylet and the neck width. A low value represents a curved stylet and a rather broader neck, while a high value represents a straighter stylet and a rather slender neck (File S8).

Principal component 5 (PC5) explains 3.4% of the overall shape variation (File S7). It is dominated by aspects of the stylet curvature and the broadness of the prothorax. A low value represents a rather straighter stylet and slender prothorax. A high value represents a rather more curved stylet and a broader prothorax (File S8).

Principal component 6 (PC6) explains 2.4% of the overall shape variation (File S7). It is dominated by aspects of the width of the head, prothorax, and the posterior trunk end. A low value represents a broader head and prothorax and a pointed trunk end. A high value represents a rather narrow head and prothorax and a wider trunk end (File S8).

Principal component 7 (PC7) explains 1.9% of the overall shape variation (File S7). It is dominated by aspects of the width of the head, prothorax, and posterior trunk end. A low value represents a rather narrow head and prothorax and a pointed trunk end. A high value represents a broader head, prothorax, and trunk end (File S8).

*Body without stylet:* The analysis resulted in seven effective PCs. Principal component 1 (PC1) explains 35.6% of the overall shape variation (File S9). It is dominated by aspects of the width of the prothorax. A low value represents a narrow prothorax, while a high value represents a slightly broader prothorax (File S10).

Principal component 2 (PC2) explains 24.1% of the overall shape variation (File S9). It is dominated by aspects of the shape of the head and trunk. A low value represents a broad head and a trunk with straight/parallel sides, while a high value represents a smaller, triangular-shaped head and a convex-shaped body (File S10).

Principal component 3 (PC3) explains 13.3% of the overall shape variation (File S9). It is dominated by aspects of the width of the head, neck, and posterior trunk end. A low value represents a broad, oval-shaped head, a narrow neck, and a pointed posterior trunk end. A high value represents a rather rectangular-shaped head, as well as a wide neck and posterior trunk end (File S10).

Principal component 4 (PC4) explains 9.6% of the overall shape variation (File S9). It is dominated by aspects of the width of the head, neck, and posterior trunk end. A low value represents a rather rectangular-shaped head, as well as a wide neck and posterior trunk end. A high value represents an oval-shaped head, a narrower neck, and a pointed posterior trunk end (File S10).

Principal component 5 (PC5) explains 3.4% of the overall shape variation (File S9). It is dominated by aspects of the trunk width. A low value represents a rather broader trunk with a pointed trunk end. A high value represents a rather narrower trunk with a wider posterior trunk end (File S10).

Principal component 6 (PC6) explains 3.1% of the overall shape variation (File S9). It is dominated by aspects of the shape of the anterior prothorax and the posterior trunk end. A low value represents a rather narrow anterior prothorax and a pointed trunk end. A high value represents a broader anterior prothorax and trunk end (File S10).

Principal component 7 (PC7) explains 2.1% of the overall shape variation (File S9). It is dominated by aspects of the head capsule shape, width of the neck, and shape of the posterior trunk end. A low value represents a smaller, triangular-shaped head, and a rather broader neck and posterior trunk end. A high value represents an oval-shaped head, a narrower neck, and a pointed posterior trunk end (File S10).

*Head, prothorax, and stylet:* The analysis resulted in six effective PCs. Principal component 1 (PC1) explains 55.4% of the overall shape variation (File S11). It is dominated by aspects of the head capsule and stylet shape. A low value represents a rather rectangular-shaped head with a small labrum; the stylet with a narrow proximal part is rather straight, broader at the distal end, and positioned on the outer part of the anterior head. A high value represents a rather bellied, oval-shaped head; the stylet with a broad proximal part is positioned more in the middle of the anterior head and tapers strongly towards the distal end (File S12).

Principal component 2 (PC2) explains 18.6% of the overall shape variation (File S11). It is dominated by aspects of the head capsule and the stylet shape. A low value represents a rather rectangular-shaped head and an elongated stylet with a broad proximal part, tapering distally. A high value represents a rather bellied, heart-shaped head and a shorter, distal broader stylet with a narrow proximal part (File S12).

Principal component 3 (PC3) explains 6.8% of the overall shape variation (File S11). It is dominated by aspects of the anterior and posterior areas. A low value represents a rather straight stylet with a small proximal part and a broader distal end, as well as a rectangular posterior end. A high value represents a rather straight stylet with a broad proximal part and pointed distal end, as well as a more rounded posterior end (File S12).

Principal component 4 (PC4) explains 5.2% of the overall shape variation (File S11). It is dominated by aspects of the anterior and posterior areas. A low value represents a rather small stylet, a tiny labrum, and a rounded posterior end. A high value represents a rather prominent stylet, a larger labrum, and a rectangular-shaped posterior end (File S12).

Principal component 5 (PC5) explains 3.0% of the overall shape variation (File S11). It is dominated by aspects of the neck width and the posterior area. A low value represents a broader neck and a rather rectangular-shaped posterior end of the prothorax. A high value represents a narrower neck and a more rounded posterior end of the prothorax (File S12).

Principal component 6 (PC6) explains 2.0% of the overall shape variation (File S11). It is dominated by aspects of the shape of the stylet, the head, and the posterior end. A low value represents a rather straight stylet with a pointed distal end, a narrow oval-shaped head transition into the neck, and a rounded posterior end of the prothorax. A high value represents a rather straight stylet with a broader distal end, a narrower-shaped head transition straight into the neck, as well as a more rectangular-shaped posterior end of the prothorax (File S12).

*Head and prothorax:* The analysis resulted in eight effective PCs. Principal component 1 (PC1) explains 35.7% of the overall shape variation (File S13). It is dominated by aspects of the anterior and posterior ends. A low value represents a smaller labrum, a narrower middle area, and a round posterior shape. A high value represents a longer labrum, a broader middle area, and a rectangular posterior shape (File S14).

Principal component 2 (PC2) explains 17.2% of the overall shape variation (File S13). It is dominated by aspects of the anterior and posterior ends. A low value represents a short or not visible labrum and a broad rectangular posterior end. A high value represents a long labrum and a narrow round posterior end (File S14).

Principal component 3 (PC3) explains 13.1% of the overall shape variation (File S13). It is dominated by aspects of the anterior end. A low value represents a small labrum. A high value represents a long labrum (File S14).

Principal component 4 (PC4) explains 8.6% of the overall shape variation (File S13). It is dominated by aspects of the anterior end, the posterior end of the head capsule, and the posterior area. A low value represents a short labrum, a head transition straight into the prothorax, and a round posterior area. A high value represents a long labrum, a head clearly separating with a rounded shape from the prothorax and a rectangular posterior area (File S14).

Principal component 5 (PC5) explains 7.0% of the overall shape variation (File S13). It is dominated by aspects of the anterior end and the posterior end. A low value represents a short labrum, a rectangular anterior end of the head, and a straight posterior end. A high value represents a long labrum, a round anterior end of the head, and a convex posterior end (File S14).

Principal component 6 (PC6) explains 5.0% of the overall shape variation (File S13). It is dominated by aspects of the anterior end and the posterior area. A low value represents a narrow labrum, a rectangular anterior end of the head, and a rectangular posterior area. A high value represents a broad labrum, a round anterior end of the head, and a round posterior area (File S14).

Principal component 7 (PC7) explains 3.0% of the overall shape variation (File S13). It is dominated by aspects of the anterior end and the area in the middle. A low value represents a short, rounded labrum and an area in the middle with two clefts, one posterior to the head and one anterior to the broad posterior area. A high value represents a long pointed labrum and an area in the middle with a single cleft (File S14).

Principal component 8 (PC8) explains 2.2% of the overall shape variation (File S13). It is dominated by aspects of the anterior end, posterior area, and the area in the middle. A low value represents a broad labrum, a round head, an area in the middle with a single cleft, and a rectangular posterior area. A high value represents a narrow labrum, a rectangular head, an area in the middle with two clefts, and a round posterior area (File S14).

*Prothorax:* The analysis resulted in seven effective PCs. Principal component 1 (PC1) explains 37.8% of the overall shape variation (File S15). It is dominated by aspects of the anterior and posterior end and the shape of the middle. A low value represents a concave anterior end, a straight rectangular posterior end, and a convex middle shape. A high value represents a convex anterior end, a round posterior end, and a concave middle with the more posterior part being broader than the anterior (File S16).

Principal component 2 (PC2) explains 22.2% of the overall shape variation (File S15). It is dominated by aspects of the anterior and posterior areas. A low value represents a broad concave posterior end getting narrower toward the middle and a rectangular anterior end. A high value represents a round convex posterior end becoming broader toward the middle forming a convex posterior half and a round anterior end (File S16).

Principal component 3 (PC3) explains 16.1% of the overall shape variation (File S15). It is dominated by aspects of the anterior half and the posterior area. A low value represents a narrow anterior end, a convex outer shape of the anterior half, and a narrow posterior area. A high value represents a broad anterior end, a concave outer shape of the anterior half, and a broad posterior area (File S16).

Principal component 4 (PC4) explains 5.3% of the overall shape variation (File S15). It is dominated by aspects of the anterior and posterior areas. A low value represents a rectangular shape of the anterior and posterior areas. A high value represents a rounded shape of the anterior and posterior area (File S16).

Principal component 5 (PC5) explains 4.9% of the overall shape variation (File S15). It is dominated by aspects of the anterior area and the posterior end. A low value represents a narrower, extended anterior end and a set-off, broader, and slightly convex posterior end. A high value represents a broad anterior end with a small extended part on the outer side and a not set-off, slightly convex posterior end (File S16).

Principal component 6 (PC6) explains 3.1% of the overall shape variation (File S15). It is dominated by aspects of the anterior and posterior end and the area in the middle. A low value represents a slightly concave anterior end and a smooth transition to the broader posterior area. A high value represents a round anterior end and a more distinct set-off broader posterior area (File S16).

Principal component 7 (PC7) explains 2.4% of the overall shape variation (File S15). It is dominated by aspects of the anterior and posterior end and the area in the middle. A low value represents a concave anterior end, a roughly rectangular posterior end, and a smooth transition from the narrower anterior to the broader posterior end. A high value represents a convex anterior end, a round posterior end, and a more distinct set-off broader posterior area (File S16).

*Trunk (with prothorax):* The analysis resulted in seven effective PCs. Principal component 1 (PC1) explains 35.1% of the overall shape variation (File S17). It is dominated by aspects of the anterior half and the posterior area. A low value represents a broad concave shape of the prothorax, a narrow shape of the posterior area of the thorax and anterior area of the abdomen, as well as a broader shape of the posterior area of the abdomen. A high value represents a narrow-elongated shape of the prothorax, a broad shape of the posterior area of the thorax and anterior area of the abdomen, as well as a narrow shape of the posterior area of the abdomen (File S18).

Principal component 2 (PC2) explains 30.5% of the overall shape variation (File S17). It is dominated by aspects of the anterior area. A low value represents a narrow shape of the prothorax and a broader shape of the mesothorax and metathorax. A high value represents a more uniform shape of the broadness of the thorax (File S18).

Principal component 3 (PC3) explains 10.6% of the overall shape variation (File S17). It is dominated by aspects of the anterior area. A low value represents a not set-off anterior region, as well as a narrow shape of the mesothorax and the anterior end of the abdomen. A high value represents a more set-off anterior end, as well as a broad shape of the mesothorax and the anterior end of the abdomen (File S18).

Principal component 4 (PC4) explains 9.2% of the overall shape variation (File S17). It is dominated by aspects of the anterior and posterior ends. A low value represents a broad concave anterior end and a broad posterior end. A high value represents a narrow pointed anterior end and a narrow spindle-shaped posterior end (File S18).

Principal component 5 (PC5) explains 3.2% of the overall shape variation (File S17). It is dominated by aspects of the thorax, the middle of the abdomen, and the area of the posterior end. A low value represents a broad shape of the thorax and the posterior area and a narrow shape of the middle of the abdomen. A high value represents a narrow shape of the thorax and the posterior area and a broad shape of the middle of the abdomen (File S18).

Principal component 6 (PC6) explains 2.3% of the overall shape variation (File S17). It is dominated by aspects of the anterior area. A low value represents a broader anterior end and a narrow mesothorax. A high value represents a narrower anterior end and a broad mesothorax (File S18).

Principal component 7 (PC7) explains 2.0% of the overall shape variation (File S17). It is dominated by aspects of the anterior end and the broadness of certain areas. A low value represents a round shape of the anterior end, a broad shape of the metathorax, a narrow shape of abdomen segment 1, and a broad shape of two areas on the posterior end. A high value represents a pointed shape of the anterior end, a narrow shape of the metathorax, a broad shape of abdomen segment 1, and a narrow shape of two areas on the posterior end (File S18).

*Trunk without prothorax:* The analysis resulted in seven effective PCs. Principal component 1 (PC1) explains 36.2% of the overall shape variation (File S19). It is dominated by aspects of the anterior end. A low value represents a straight and a high value a more rounded anterior end (File S20).

Principal component 2 (PC2) explains 31.7% of the overall shape variation (File S19). It is dominated by aspects of the anterior end. A low value represents a convex shape of the anterior end of the mesothorax and a high value represents a more concave shape (File S20).

Principal component 3 (PC3) explains 12.2% of the overall shape variation (File S19). It is dominated by aspects of the anterior and posterior ends. A low value represents a narrow shape of the anterior end and a pointed posterior end. A high value represents a broader shape of the anterior end and a larger extension antero-laterally, as well as a more rounded shape of the posterior end (File S20).

Principal component 4 (PC4) explains 4.4% of the overall shape variation (File S19). It is dominated by aspects of different segments of the body. A low value represents a narrow shape of the mesothorax and the posterior segments, a broader shape of the area between the thorax and abdomen, as well as a broader shape of a part in the middle of the abdomen. A high value represents a broader shape of the mesothorax and the posterior segments, a narrow shape of the area between the thorax and abdomen, as well as a narrow shape of a part in the middle of the abdomen (File S20).

Principal component 5 (PC5) explains 3.4% of the overall shape variation (File S19). It is dominated by aspects of the area between the thorax and abdomen. A low value represents a narrow shape of the area between the thorax and abdomen and a high value represents a broad shape of the area between the thorax and abdomen (File S20).

Principal component 6 (PC6) explains 2.8% of the overall shape variation (File S19). It is dominated by aspects of the anterior part of the abdomen and the shape of the thorax. A low value represents a narrow shape of the anterior part of the abdomen and a round shape of the thorax. A high value represents a broad shape of the anterior part of the abdomen and a more rectangular shape of the thorax (File S20).

Principal component 7 (PC7) explains 1.8% of the overall shape variation (File S19). It is dominated by aspects of the anterior part and posterior end. A low value represents a rectangular shape of the antero-lateral area and a pointed posterior end. A high value represents a more rounded antero-lateral area and a more rounded posterior end (File S20).

The detailed files resulting from the shape analyses can be found in File S21.

## 4. Discussion

### 4.1. Identity of the New Specimens

The head of one specimen preserved in NHMD 115214 has been figured before [86]. The piece has been mentioned to include three such specimens and these were interpreted as larvae of Psychopsidae (silky lacewings). Later authors were unsure about this interpretation [16,87], but could neither support nor reject it. We can here show that these are stage 1 larvae of Nevrorthidae and that the piece includes more than three specimens. In general, lacewing larvae usually only occur closely together shortly after hatching, so this is another indication of these specimens being stage 1 larvae. The same seems to be the case in the 31 specimens in PED 0871.

For all new specimens reported here, the interpretation of them as dragon lacewing larvae is relatively clear. The arrangement of the mouthparts immediately indicates that the specimens are larvae of lacewings: no separate maxillae are apparent as they are functionally coupled to the mandibles and lack palps; of the labium only the palps are apparent; all mouthparts are strongly forward oriented (prognathous). The shape of the stylets is very indicative, as stylets that are proximally straight and distally curving are very uncommon. Besides in Nevrorthidae, this morphology is only known in representatives of the extinct group *Ankyloleon* [88,89], and other aspects of the morphology of the new larvae are very different from those of *Ankyloleon*. Hence, an interpretation of the larvae as dragon lacewings is the most parsimonious one.

### 4.2. The Assumed Stasis of Dragon Lacewing Larvae

As pointed out, it has been assumed that dragon lacewings remained more or less unchanged over geological times. Yet, already, the recently reported larvae from the Jurassic [43] represented an indication that dragon lacewing larvae of the past might have been different from their modern counterparts, as these larvae had an unusually elongated neck and a less prominent labrum.

**Figure 33:**
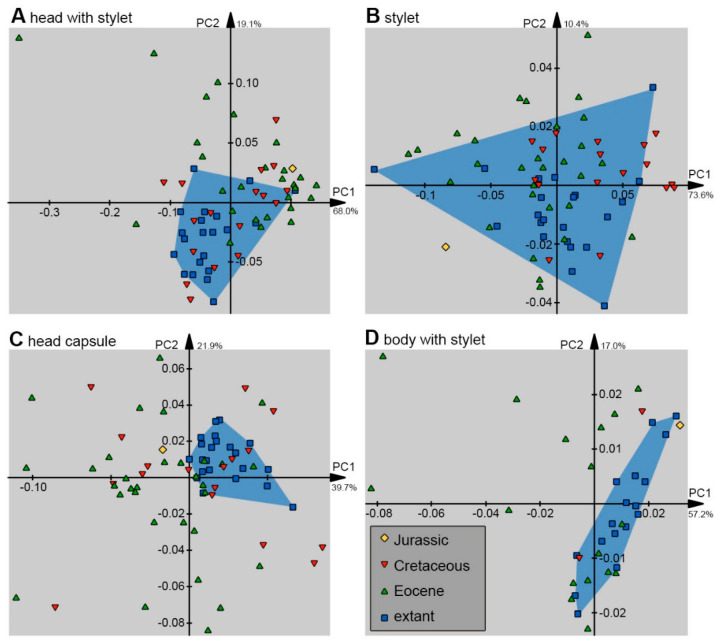
Morphospaces represented by scatter plots of the principal components (PCs) 1 and 2 of different morphological structures.

**Figure 34:**
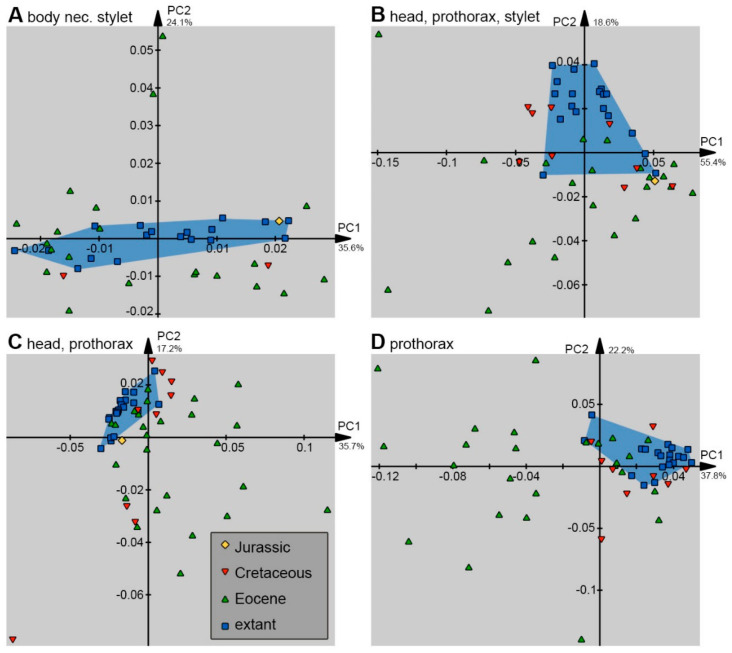
Morphospaces represented by scatter plots of the principal components (PCs) 1 and 2 of different morphological structures, continued.

The quantitative analysis performed here further supports this view. In all analysed structures, the area occupied by modern forms is comparably small. Numerous fossils plotting outside this area indicate that they differ from the modern forms. This difference is least expressed in the shape of the stylets (Figure 33B). Therefore, the shape of the stylets is indeed indicative of the fossils being dragon lacewings (see above). This result for the stylets partly influences some of the other analyses. The combined shape of the head with stylets (Figure 33A) shows fewer differences than the head capsules alone (Figure 33C); the combined shape of the head, prothorax, and stylets (Figure 34B) shows fewer differences than the head and prothorax without stylets (Figure 34C).

**Figure 35:**
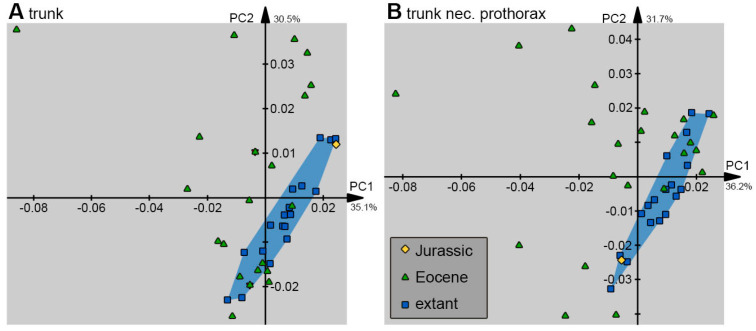
Morphospaces represented by scatter plots of the principal components (PCs) 1 and 2 of different morphological structures, continued.

The single Jurassic specimen that could be included in the analysis plots in most cases outside of the area occupied by modern forms (although not always far, Figure 34B,C, and in few cases not at all, Figure 34A and Figure 35), indicating its morphological difference (Figure 33). Also, many Cretaceous, and especially many Eocene specimens, plot quite outside the area of the modern forms. For the Eocene specimens, part of this pattern may be explained by the fact that only for this period, we have stage 1 larvae; even among the extant material, no stage 1 larvae are known. Yet, also quite some of the later-stage individuals differ in certain aspects from their modern relatives. In particular, the head is often shorter in relation to the stylets and in many cases has a less expressed forward-projecting labrum. Such shorter heads and labra seem clearly absent in the modern fauna. Also, concerning the trunk, there are morphologies present in the fossils, which are absent among the modern forms. As already noted earlier [50], some of the fossil larvae have rather short trunks that appear less elongated, giving the possible impression of a prepupa, but are the wrong size, i.e., being simply too small to already represent a prepupa. In light of the new finds, these may simply represent a morphological variation that is absent in the modern fauna.

We can conclude that dragon lacewing larvae indeed have changed over time (that they do not represent living fossils has already been elaborated in [50]). The expanded data set now also shows a clear loss of morphological diversity for this group over time.

### 4.3. Loss of Diversity in Other Groups of Lacewings

Earlier studies have already shown losses in diversity for long-nosed antlions, i.e., the larvae of silky lacewings (Psychopsidae; [87]) and larvae of split-footed lacewings (Nymphidae; [29]). In other groups, no real change was apparent [90], or the data were inconclusive [91,92]. Yet, in a few groups (although a certain loss may have been recognised), an overall later diversification could be identified [30,90,93]. This complex pattern is also difficult to interpret as the data sets are still too incomplete, yet a net loss of overall diversity larvae of Neuroptera remains unquestioned [7]. Even larger sub-sets of data seem so far not yet saturated [31]; hence, it is to be expected that even more new morphologies remain to be found in the fossil record.

Nevrorthidae, so far, did not show a clear pattern of loss. Expanding the data set here has changed this situation (the data set size has been more than doubled since our last study; [50]); also, this group seems to have undergone a decline. It still remains unclear whether the seemingly highest morphological diversity in the Eocene is a true signal or an artefact due to the larger sub-sample size. It remains to be awaited to find stage 1 individuals in the Cretaceous (which should be expected) as well as more larvae in the Jurassic.

### 4.4. A Brief Look on Palaeoecology

First, it is important to reiterate that not all extant reports of dragon lacewing larvae suggest an aquatic lifestyle. Riek ([53] p. 487) reported one larva that he found in moist leaf litter. Therefore, it cannot be taken as given that all fossil dragon lacewing larvae were aquatic. Yet, it seems clearly likely that they also lived at least in very moist environments, and it is possible that some of them lived in freshwater, at least the fossils preserved in amber, as they are known in size ranges comparable to those of the modern forms. Still, the fact that some of the amber fossils are less slender than their modern counterparts and have different head and labrum shapes indicates that they were hunting in slightly different ways than their modern relatives. They may also have searched for different prey.

More tricky to interpret are the recently reported Jurassic larvae [43]. These are true giants roughly 40 mm in size, more than three times the maximum size of the largest known extant larva (about 13 mm; [52]). Extant dragon lacewing larvae lack gill structures; also, in the fossils, no indications of such structures are known. This lack seems unproblematic for the modern larvae, yet these are very slender and not very large, and hence, gas exchange over the body surface may well be sufficient, especially as they are living in highly oxygenated running waters. The large fossils are in a quite different size range and appear not as slender as the modern forms, besides the neck, which is more slender than in any other form. Hence, at this size, we could expect that they should possess gills when living in water. This absence of gills does not exclude that these fossils were aquatic. Some larvae of predaceous diving beetles (Dytiscidae) reach even larger sizes than the Jurassic fossils, resembling the overall body shape, and some do not possess gills either. This leaves the possibility that also these large larvae were aquatic.

The comparison to larvae of predaceous diving beetles may indeed provide a good comparison for the Jurassic fossils (though these beetle larvae cannot breathe underwater, but take in oxygen at the surface). The large predaceous diving beetle larvae, better known as water tigers, are top predators consuming everything in their range, including fishes, tadpoles, or even adult amphibians [94]. We can expect that large-sized lacewing larvae, which are (almost) all known to be effective predators and venomous, indeed were able to fulfil a similar ecological role. For food web reconstructions, such aspects will have to be considered. So far, direct interactions of fossil lacewing larvae with their prey are practically absent; only a single piece from Baltic amber has preserved an aphidlion together with some aphids in the same piece [95]. Therefore, also some more indirect comparisons as discussed here are necessary to gain at least some insights into the feeding ecology of the extinct animals.

## 5. Outlook

The study of Haug et al. [50] significantly expanded the known record of dragon lacewing larvae; the study of Du et al. [43] expanded the time and size range of dragon lacewing larvae; the present study more than doubled the number of available specimens. Although we can not test for saturation in the same manner as in other cases [31], it should be clear that the data set is not yet saturated, as stage 1 larvae are only known from a single time slice, and only a single specimen from the Jurassic could be included into the quantitative analysis. It is therefore to be expected that further expanding the data set may still change the picture.

Yet, already the still incomplete data set shows that the anticipated stasis of dragon lacewing larvae is not supported by quantitative data. Instead, dragon lacewings line up with other lineages of lacewings, having declined over time concerning the morphological diversity of their larval stages.

## Figures and Tables

**Figure 21:**
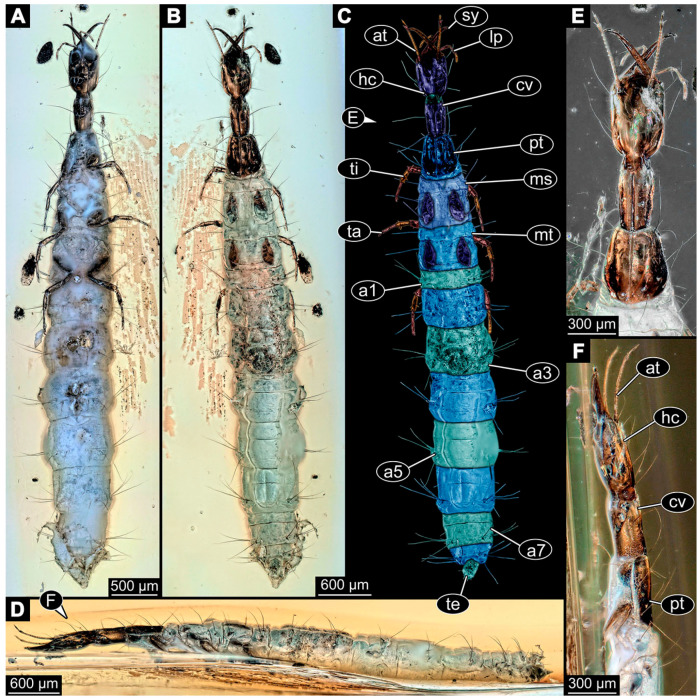
Nevrorthidae; fossil larva preserved in Baltic amber; specimen 6703 (CCHH 1387-1); ring light. (**A**–**D**). Overviews. (**A**). Ventral view. (**B**). Dorsal view. (**C**). Colour-marked version of (**B**). (**D**). Lateral view, image flipped. (**E**,**F**). Anterior body region. (**E**). Dorsal view. (**F**). Lateral view, image flipped. Abbreviations: a1–7 = abdomen segments 1–7; at = antenna; cv = cervix (neck); hc = head capsule; lp = labial palp; ms = mesothorax; mt = metathorax; pt = prothorax; sy = stylet; ta = tarsus; te = trunk end; ti = tibia.

**Figure 22:**
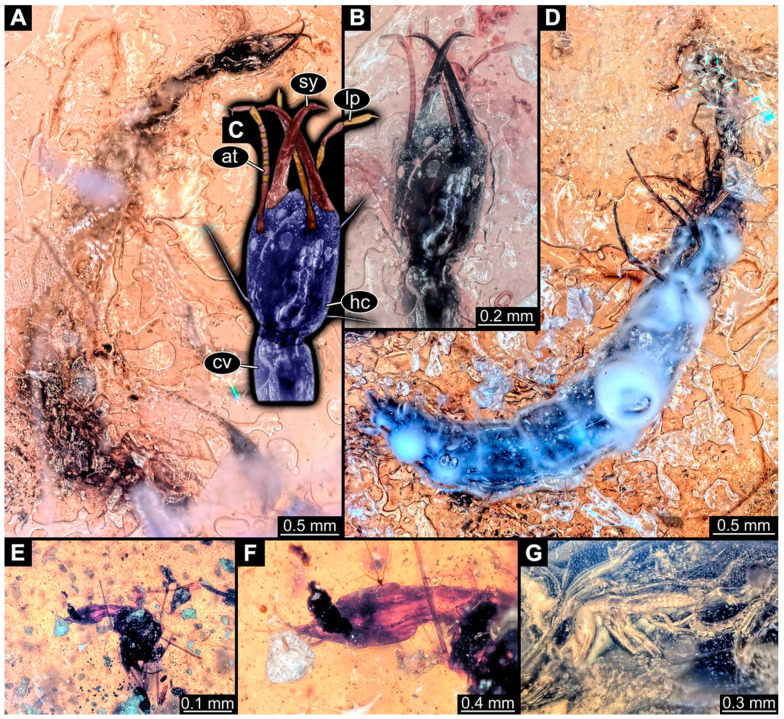
Nevrorthidae; fossil larvae preserved in Baltic amber and Kachin amber. (**A**–**D**). Specimen 6705 (PED 0792), Baltic amber. (**A**). Lateral view, right. (**B**). Close-up of head. (**C**). Colour-marked version of (**B**). (**D**). Lateral view, left. (**E**,**F**). Specimen 6713 (PED 2001), Kachin amber. (**E**). Dorsal view. (**F**). Close-up of head. (**G**). Specimen 6704 (SMF Be 2192), Baltic amber; lateral view, left. (**A**,**B**,**D**,**F**). Ring light. (**E**,**G**). Mixed light. Abbreviations: at = antenna; cv = cervix (neck); hc = head capsule; lp = labial palp; sy = stylet.

**Figure 23:**
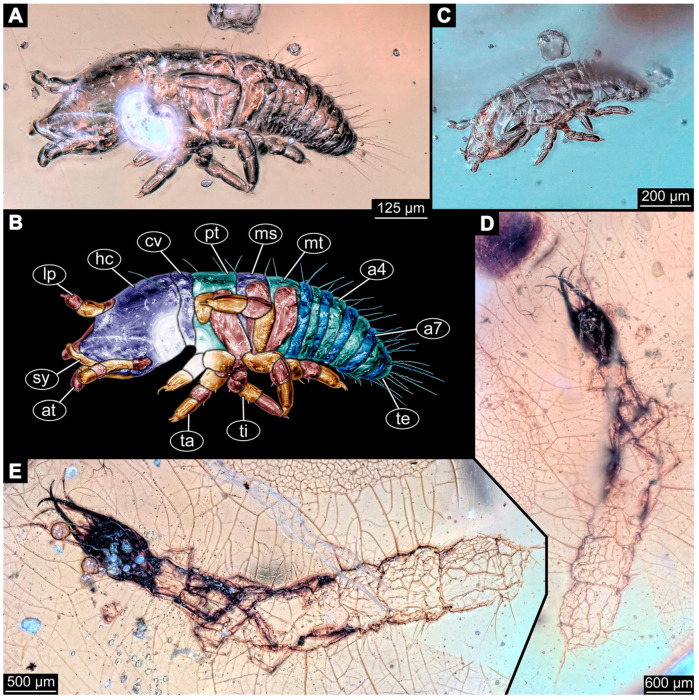
Nevrorthidae; fossil larvae preserved in Baltic amber and Kachin amber. (**A**–**C**). Specimen 6706 (PED 1379), Baltic amber. (**A**). Ventral view. (**B**). Colour-marked version of (**A**). (**C**). Dorsal view, image flipped. (**D**,**E**). Specimen 6716 (PED 2662), Kachin amber. (**D**). Ventral view. (**E**). Dorsal view. (**A**). Cross-polarised light. (**C**–**E**). Ring light. Abbreviations: a4–7 = abdomen segments 4–7; at = antenna; cv = cervix (neck); hc = head capsule; lp = labial palp; ms = mesothorax; mt = metathorax; pt = prothorax; sy = stylet; ta = tarsus; te = trunk end; ti = tibia.

## Data Availability

All data from this study are available in this paper and the associated papers.

## Supplementary Materials

The following supporting information can be downloaded at: https://www.mdpi.com/2075-4450/14/9/749/s1 and https://doi.org/10.5281/zenodo.7937268 (accessed on 5 September 2023), Supplementary File S1. Outcome of the principal component analysis of heads and stylets. File S2. Graphical representation of the factor loadings of the principal component analysis of heads and stylets. File S3. Outcome of the principal component analysis of stylets. File S4. Graphical representation of the factor loadings of the principal component analysis of stylets. File S5. Outcome of the principal component analysis of heads. File S6. Graphical representation of the factor loadings of the principal component analysis of heads. File S7. Outcome of the principal component analysis of bodies with stylets. File S8. Graphical representation of the factor loadings of the principal component analysis of bodies with stylets. File S9. Outcome of the principal component analysis of bodies without stylets. File S10. Graphical representation of the factor loadings of the principal component analysis of bodies without stylets. File S11. Outcome of the principal component analysis of heads, prothorax, and stylets. File S12. Graphical representation of the factor loadings of the principal component analysis of heads, prothorax and stylets. File S13. Outcome of the principal component analysis of heads and prothorax. File S14. Graphical representation of the factor loadings of the principal component analysis of heads and prothorax. File S15. Outcome of the principal component analysis of prothorax. File S16. Graphical representation of the factor loadings of the principal component analysis of prothorax. File S17. Outcome of the principal component analysis of trunk with prothorax. File S18. Graphical representation of the factor loadings of the principal component analysis of trunk with prothorax. File S19. Outcome of the principal component analysis of trunk without prothorax. File S20. Graphical representation of the factor loadings of the principal component analysis of trunk without prothorax. File S21. Files resulting from the shape analyses, including chain codes, aligned shapes, and principal component analyses. Figure 1, Figure 2, Figure 3, Figure 4, Figure 5, Figure 6, Figure 7, Figure 8, Figure 9, Figure 10, Figure 11, Figure 12, Figure 13, Figure 14, Figure 15, Figure 16, Figure 17, Figure 18, Figure 19, Figure 20, Figure 21, Figure 22, Figure 23, Figure 24, Figure 25, Figure 26, Figure 27, Figure 28, Figure 29, Figure 30, Figure 31, Figure 32, Figure 33, Figure 34 and Figure 35 in high resolution are available in the Supplementary Materials.
