# Supplementary figures and images for: The Morphological Diversity of Dragon Lacewing Larvae (Nevrorthidae, Neuroptera) Changed More over Geological Time Scales Than Anticipated
Source: Insects. 2023 Sep 6;14(9):749. doi: 10.3390/insects14090749 (PMC10649721; doi:10.3390/insects14090749)

-2S.D.

Mean

+2S.D.

PC1

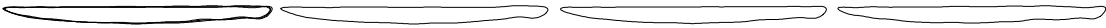

PC2

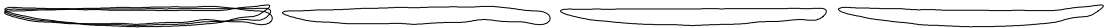

PC3

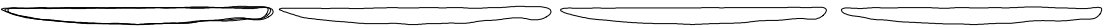

PC4

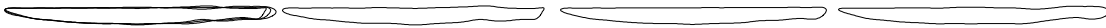

PC5

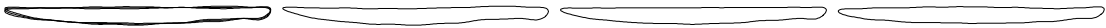

-2S.D.

Mean

+2S.D.

PC6

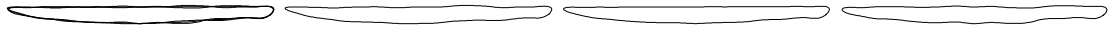

PC7

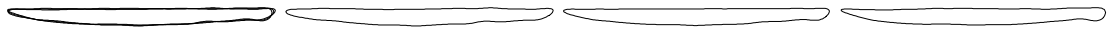

Supplement: Supplementary file 1 [file insects-14-00749-s001.zip › Supplement Mengel/S10.pdf]

-2S.D.

Mean

+2S.D.

PC1

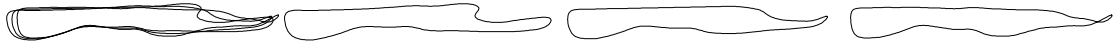

PC2

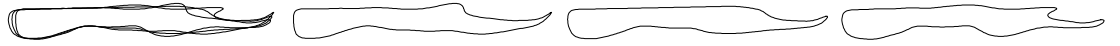

PC3

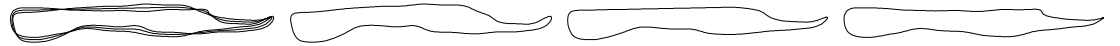

PC4

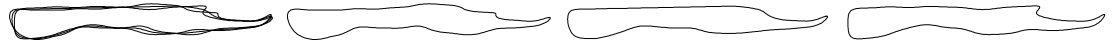

PC5

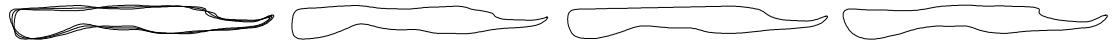

-2S.D.

Mean

+2S.D.

PC6

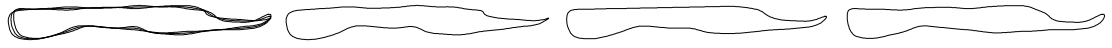

Supplement: Supplementary file 1 [file insects-14-00749-s001.zip › Supplement Mengel/S12.pdf]

-2S.D.

Mean

+2S.D.

PC1

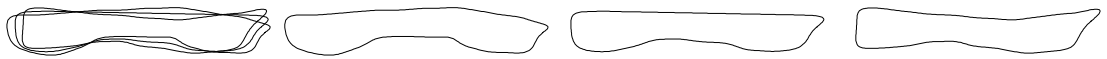

PC2

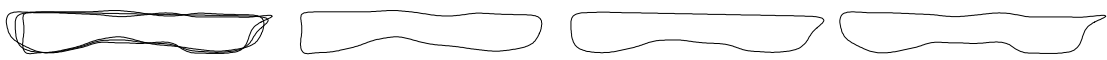

PC3

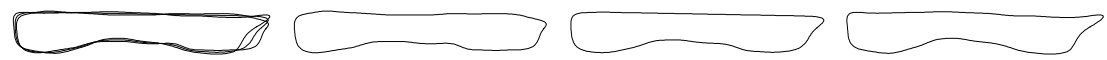

PC4

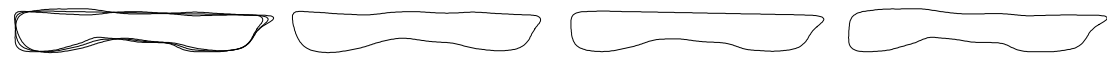

PC5

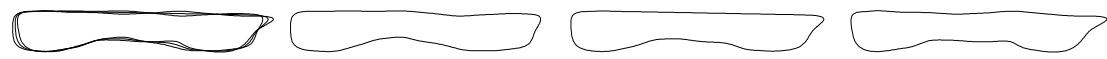

-2S.D.

Mean

+2S.D.

PC6

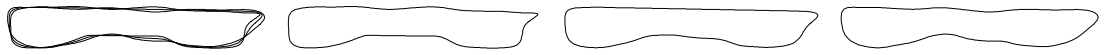

PC7

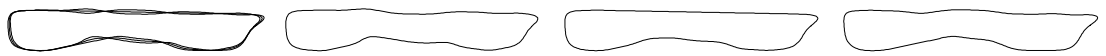

PC8

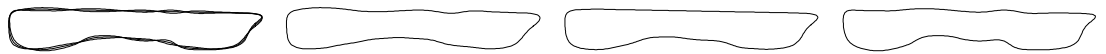

Supplement: Supplementary file 1 [file insects-14-00749-s001.zip › Supplement Mengel/S14.pdf]

-2S.D.

Mean

+2S.D.

PC1

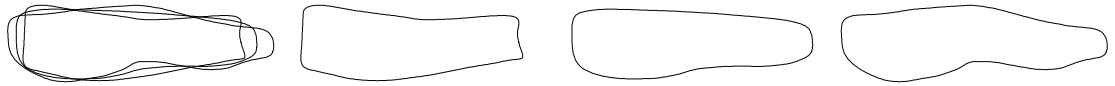

PC2

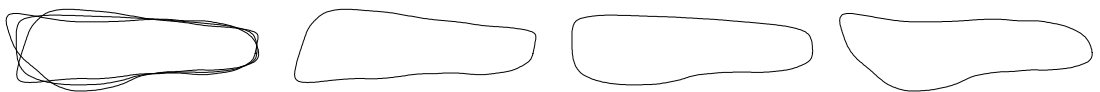

PC3

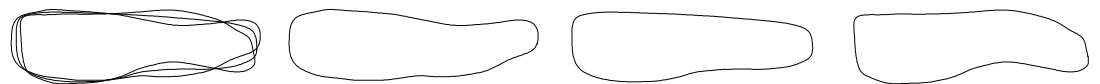

PC4

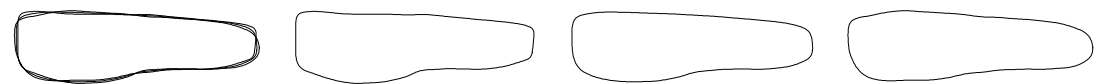

PC5

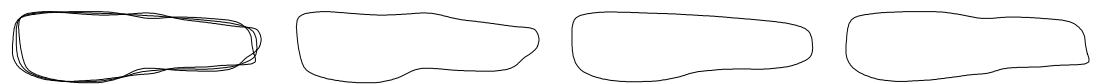

-2S.D.

Mean

+2S.D.

PC6

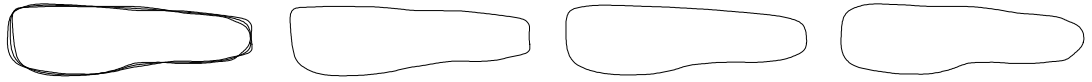

PC7

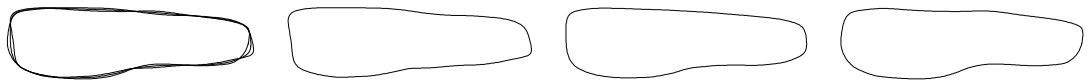

Supplement: Supplementary file 1 [file insects-14-00749-s001.zip › Supplement Mengel/S16.pdf]

-2S.D.

Mean

+2S.D.

PC1

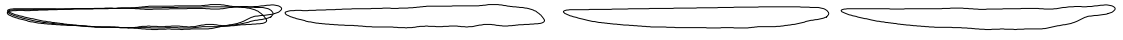

PC2

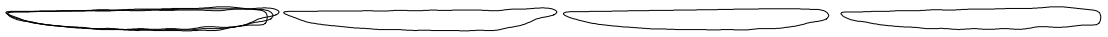

PC3

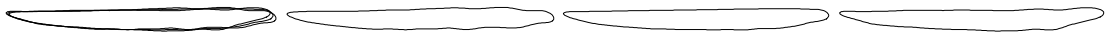

PC4

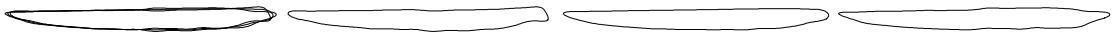

PC5

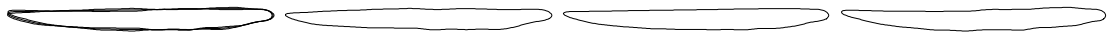

-2S.D.

Mean

+2S.D.

PC6

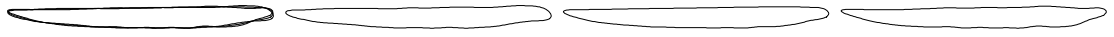

PC7

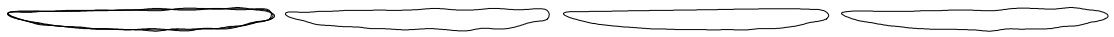

Supplement: Supplementary file 1 [file insects-14-00749-s001.zip › Supplement Mengel/S18.pdf]

-2S.D.

Mean

+2S.D.

PC1

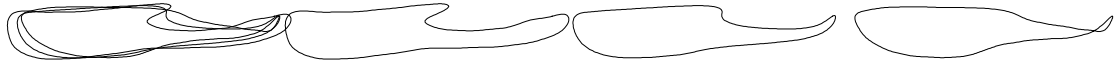

PC2

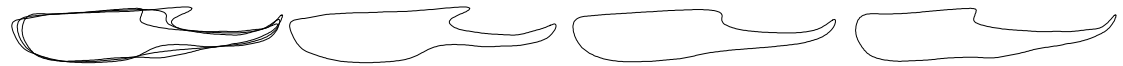

PC3

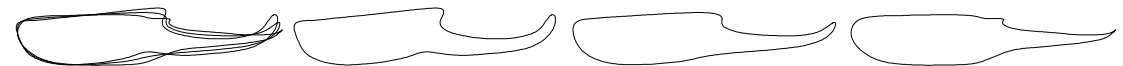

PC4

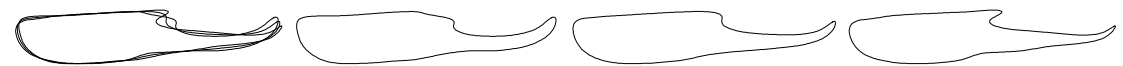

Supplement: Supplementary file 1 [file insects-14-00749-s001.zip › Supplement Mengel/S2.pdf]

-2S.D.

Mean

+2S.D.

PC1

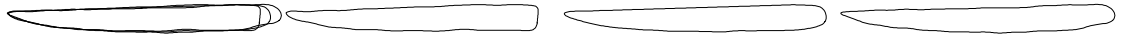

PC2

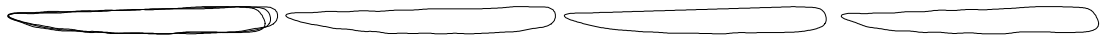

PC3

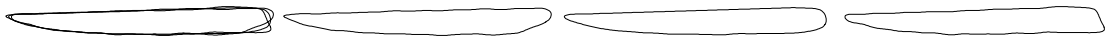

PC4

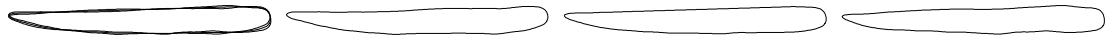

PC5

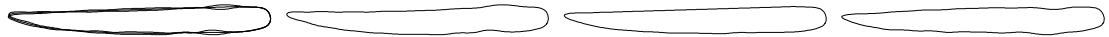

-2S.D.

Mean

+2S.D.

PC6

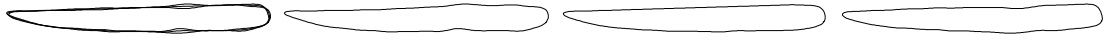

PC7

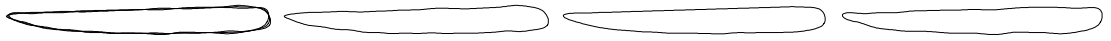

Supplement: Supplementary file 1 [file insects-14-00749-s001.zip › Supplement Mengel/S20.pdf]

-2S.D.

Mean

+2S.D.

PC1

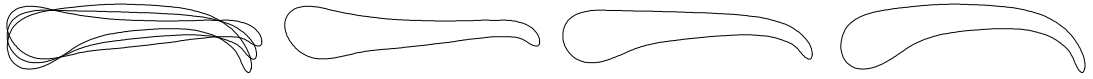

PC2

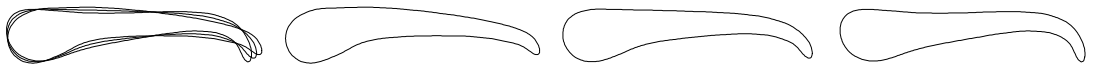

PC3

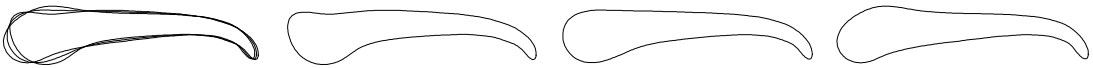

PC4

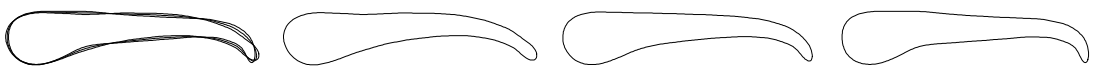

Supplement: Supplementary file 1 [file insects-14-00749-s001.zip › Supplement Mengel/S4.pdf]

-2S.D.

Mean

+2S.D.

PC1

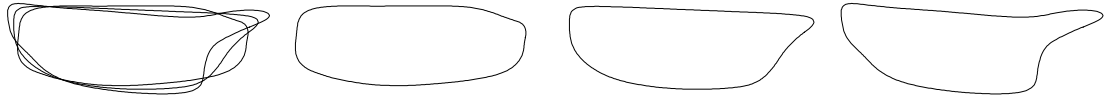

PC2

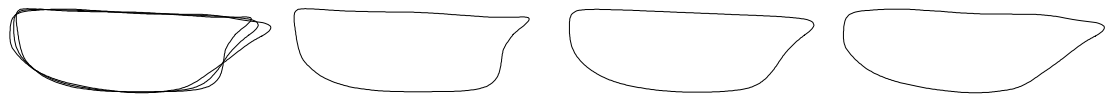

PC3

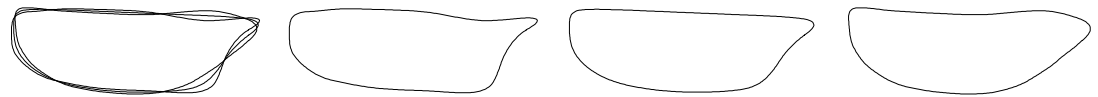

PC4

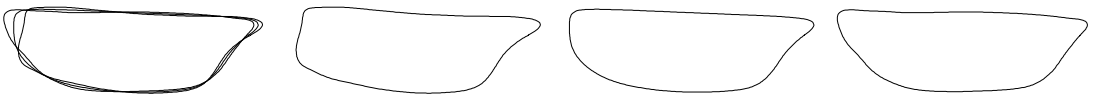

PC5

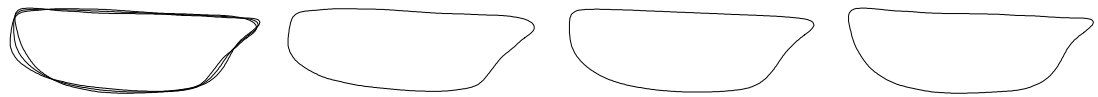

-2S.D.

Mean

+2S.D.

PC6

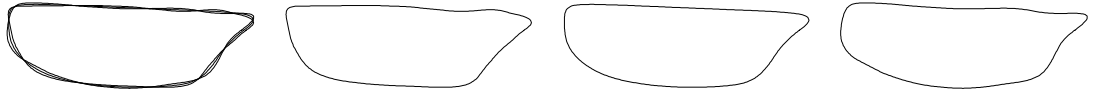

PC7

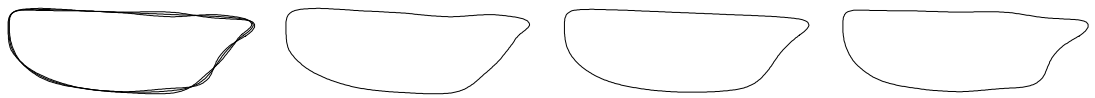

Supplement: Supplementary file 1 [file insects-14-00749-s001.zip › Supplement Mengel/S6.pdf]

-2S.D.

Mean

+2S.D.

PC1

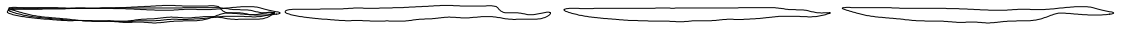

PC2

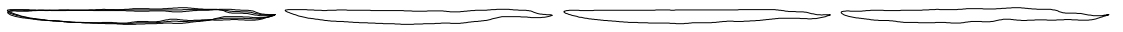

PC3

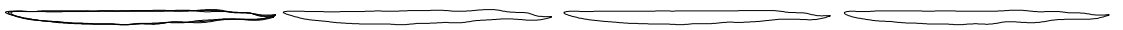

PC4

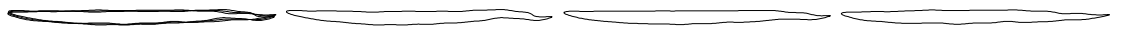

PC5

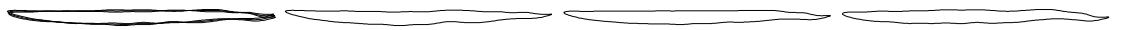

-2S.D.

Mean

+2S.D.

PC6

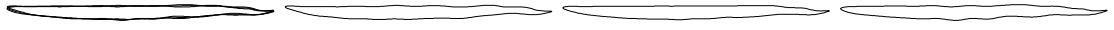

PC7

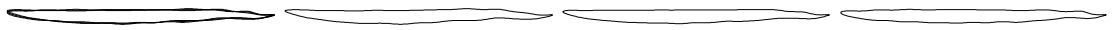

Supplement: Supplementary file 1 [file insects-14-00749-s001.zip › Supplement Mengel/S8.pdf]
